# Inference for the treatment effect in staircase designs with continuous outcomes: a simulation study

**DOI:** 10.1186/s12874-025-02567-5

**Published:** 2025-05-10

**Authors:** Ehsan Rezaei-Darzi, Kelsey L. Grantham, Andrew B. Forbes, Jessica Kasza

**Affiliations:** School of Public Health and Preventive Medicine, Monash University, Melbourne, Australia

**Keywords:** Cluster randomised trials, Incomplete design, Intracluster correlation, Stepped wedge

## Abstract

**Background:**

Staircase designs are incomplete stepped wedge designs that, unlike standard stepped wedge designs, require clusters to contribute data for only a limited number of trial periods. Previous work has provided formulae based on asymptotic results for the calculation of the power of staircase designs to detect treatment effects of interest.

**Methods:**

We conduct a simulation study to assess the finite sample performance of these formulae, and the impact of misspecifying the correlation structure when analysing data from staircase designs on inference for the treatment effect, under a range of realistic trial settings. This study focuses on basic staircase designs with one control period followed by one intervention period in each sequence. We simulate staircase trial datasets with continuous outcomes and a repeated cross-sectional measurement scheme under exchangeable and block-exchangeable intracluster correlation structures, and then fit linear mixed models with linear and categorical time period effects. For settings with a small number of clusters, Kenward-Roger and Satterthwaite small-sample corrections are applied. Comparisons are made between nominal and observed Type I error rates, and theoretically-derived study power and empirical power. The impact on inference for the treatment effect when misspecifying the intracluster correlation structure is assessed through considering performance metrics including bias and 95% confidence interval coverage.

**Results:**

Data analysis assuming an exchangeable correlation structure and application of the Satterthwaite correction controls Type I error well when the correlation structure is correctly specified, and there are a sufficient number of clusters. For the true block-exchangeable model, when fitting the correct model with the Satterthwaite correction, the observed Type I error (empirical power) can be higher (lower) than the nominal (i.e., theoretical) value when there is only 1 cluster per sequence, but otherwise, it aligns well with the nominal (theoretical) value. Misspecification of the correlation structure (fitting an exchangeable model when the true structure is block-exchangeable) can lead to inflated Type I error and poor confidence interval coverage.

**Conclusions:**

Staircase designs with one cluster per sequence should be used with caution. Additionally, using a correlation structure that allows for decay is preferable for making valid inferences for the estimation of the treatment effect.

**Supplementary Information:**

The online version contains supplementary material available at 10.1186/s12874-025-02567-5.

## Introduction

**Fig. 1:**
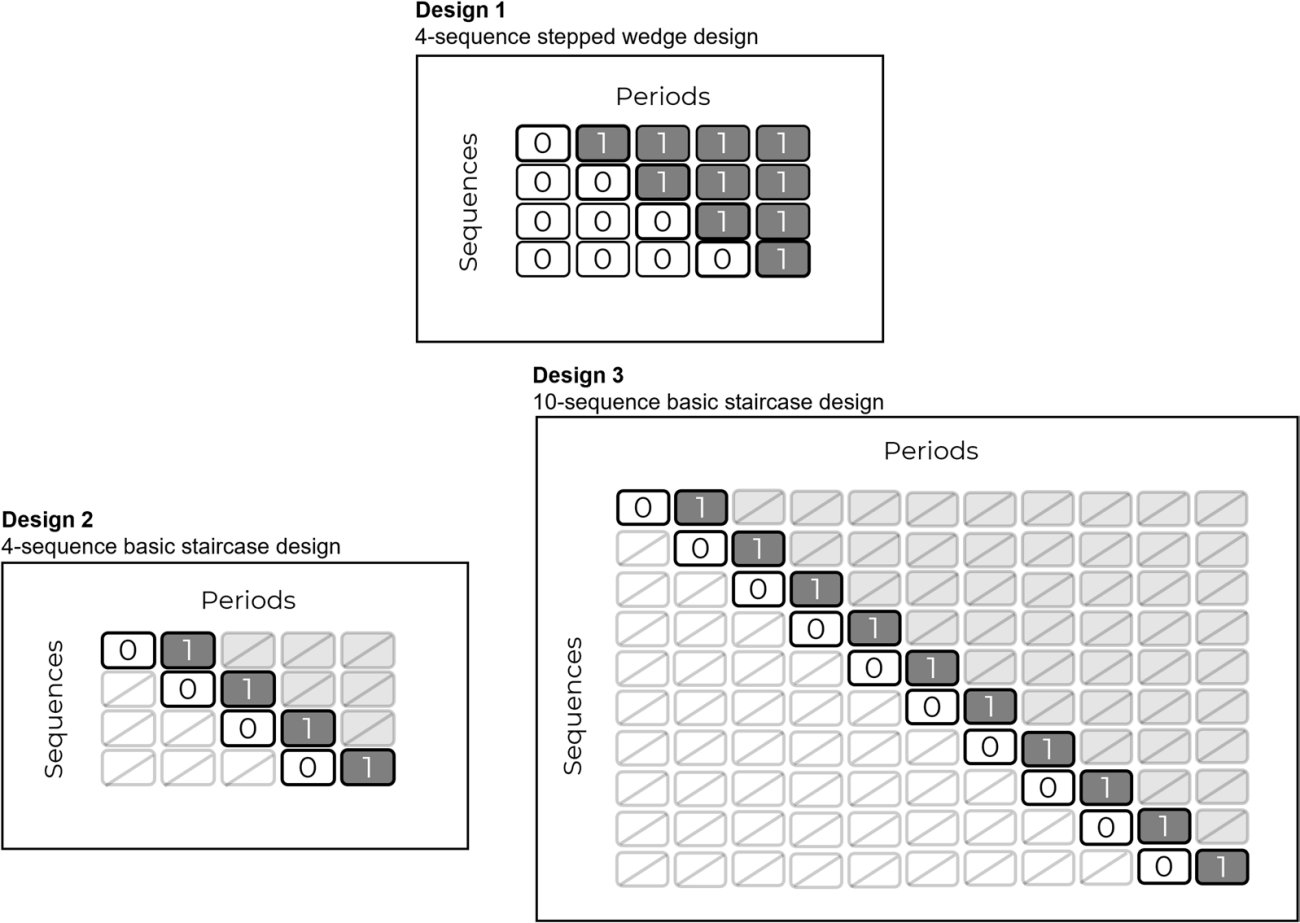
Design 1: Design schematic of a 4-sequence, 5-period standard stepped wedge design. Design 2: Design schematic of a 4-sequence basic staircase design with one period pre- and one period post intervention. Design 3: Design schematic of a 10-sequence basic staircase design with one period pre- and one period post intervention. In each design, each sequence may have a different number of clusters, which can be equal or unequal; in this paper we consider variants with equal numbers of clusters assigned to each sequence

Longitudinal cluster randomized trials, such as the designs shown in Figure 1, involve clusters switching between control and intervention conditions over time. The standard stepped wedge design (Figure 1, Design 1) requires all clusters to start in the control condition, then switch to the intervention condition at staggered times throughout the trial [1]. This design is highly appealing as it enables a phased implementation of the intervention across participating clusters, ensuring that all clusters ultimately receive the intervention [2]. In the standard stepped wedge design clusters serve as their own controls; however, gathering data from every cluster in each period may be costly or impractical [3]. Previous studies have demonstrated that when low-information cluster-period cells are removed from a complete stepped wedge design, incomplete stepped wedge designs which include only the cluster-period cells near the treatment switches and the off-diagonal corners of the design often have high statistical efficiency [4, 5].

One particular variant of incomplete stepped wedge designs is the staircase design, with a stair-like pattern of steps along the main diagonal of a stepped wedge design, where the intervention is staggered across clusters in the same manner as with the stepped wedge (Figure 1, Designs 2 and 3). Staircase designs are a pragmatic alternative to stepped wedge designs with sequences that include a limited number of pre-switch control and post-switch intervention periods, offering several practical benefits while minimising the data collection burden [6]. For example, when implementing a school-based intervention aimed at improving self-regulation in Australian Aboriginal children with foetal alcohol spectrum disorder, the researchers noted that the stepped wedge design would have placed too great a data-collection burden on the schools and their students [7]. They also noted that data collection for the entire trial duration was not possible given the limited available trial budget. These researchers instead implemented a staircase design. Several studies have explored how staircase designs, or designs resembling staircases, can offer adequate statistical power for estimating treatment effects [4, 8, 9]. Although some trials with staircase-like designs have already been conducted [10–14], theoretical results for staircase designs have only recently been developed: Grantham et al. [6] developed formulae to enable sample size and power calculations for staircase designs. This work focused on the asymptotic properties of the generalised least squares estimator of the treatment effect in staircase trial design. The finite-sample performance of these sample size formulae and the robustness of conclusions about the treatment effect to the violation of model assumptions, particularly regarding the intracluster correlation structure, should also be assessed. Since the properties of these designs are not yet fully understood, this paper evaluates whether the observed Type I error and empirical power align with their theoretical values, and examines the finite sample performance when the intracluster correlation structure is misspecified. Given the large variety of potential staircase designs, our attention is centred on a specific subset known as “basic staircase” designs [6], which place minimal data collection burden on clusters and can provide a basis for generalising findings to other staircase design variants. These designs feature only one pre- and post-switch measurement period in each sequence, with each sequence commencing data collection in a different trial period (Figure 1, Designs 2 and 3). Our first aim assesses the finite sample performance of linear mixed models-based analysis of data from basic staircase designs.

In the context of longitudinal cluster randomised trials, linear mixed models are often assumed for continuous outcomes [15]. For these models, a simplifying assumption is often made that all correlations between participants’ outcomes within the same cluster are equal, regardless of the timing of the measurements [1]. This assumption is encoded in the model through the inclusion of a cluster-level random effect. By introducing an additional random effect for cluster-periods (i.e. cells in the Fig. 1 schematic diagrams), the assumption of constant intracluster correlation can be relaxed, enabling the correlation between participants’ outcomes within the same cluster and period to differ from the correlation between participants’ outcomes within the same cluster but across different periods, known as constant between-period intracluster correlation [16–18]. This structure assumes that between-period intracluster correlations (ICCs) are constant across time. These two widely used correlation structures are also known as the exchangeable and block-exchangeable structures. A more complex intracluster correlation structure is the discrete-time decay correlation structure that models the correlation between participants'outcomes as dependent on the time between their measurement periods, with the correlation decreasing as the time gap between measurements increases [19]. However, in basic staircase designs (with only two measurement periods per sequence), the discrete-time decay correlation structure is equivalent to the block-exchangeable correlation structure.

When analysing data from stepped wedge trials, it is important to correctly specify the intracluster correlation structure, as demonstrated by Kasza and Forbes [20]. They showed that misspecification can lead to confidence intervals for the treatment effect being too wide or too narrow, depending on the particular form of the misspecification. Ouyang et al. [21] investigated the performance of a range of robust variance estimators in the presence of misspecification of the intracluster correlation structure when analysing data from stepped wedge designs and similarly showed that inference can be impacted when correlation structures are misspecified. Ji et al. [22] found that inference for the treatment effect in stepped wedge designs using linear mixed models is sensitive to model misspecification, particularly when cluster-period random effects are omitted resulting in potentially severe Type I error rate inflation. Other researchers have also investigated the impact of omitting random treatment effects in the analysis of data from stepped wedge designs, via theory [23, 24] and via simulation [25]. Here our second aim is to investigate how misspecification of the intracluster correlation structure affects observed Type I error and confidence interval coverage in basic staircase designs.

The cluster randomised trials literature often highlights concerns about the validity of sample size calculations and data analysis when standard methods are employed in trials with a “small” number of clusters [1, 26–28]. Trials with a small number of clusters can result in inflated Type I error rates when using mixed models [28]. Most studies have recommended that trials include a minimum of $30$ to $40$ clusters to maintain a Type I error rate of $5\%$ with the use of mixed models [29]. However, this number can vary greatly across different study designs. In the CONSORT extension for stepped wedge cluster randomised trials, using small-sample corrections in the analysis of stepped wedge designs when applicable was recommended (item 12a) [30]. In situations with small numbers of clusters, using maximum likelihood (ML) or restricted maximum likelihood (REML) estimation to estimate parameters of linear mixed models can underestimate the standard errors for the intervention effect [31]. Typically, in large-sample settings, test statistics such as the Wald test statistic are assumed to follow a standard normal distribution. However, in smaller samples, assuming a t-distribution for test statistics and accurately estimating the degrees of freedom becomes crucial because asymptotic results may not hold [32]. In the context of mixed models, small-sample corrections often involve adjustments to the degrees of freedom for the Wald test, like those proposed by Satterthwaite or by Kenward and Roger [33, 34]. Overestimating the degrees of freedom is likely to result in a liberal test with an inflated Type I error rate, while underestimating the degrees of freedom can lead to a conservative test, potentially reducing power [35]. Given that estimation of model parameters when there are small numbers of clusters and the application of small-sample corrections in the analysis of data have not been investigated for staircase designs, we evaluate the benefits of applying the Satterthwaite and Kenward-Roger corrections.

In this paper we consider basic staircase designs with a continuous outcome, varying the number of unique treatment sequences, clusters per sequence, periods, and participants per cluster in each period. For each design, continuous outcomes are simulated under two intracluster correlation structures: exchangeable and block-exchangeable. We consider various within-period intracluster correlation values and, for the block-exchangeable structure, different degrees of decay in the intracluster correlation. REML-based estimation is used to estimate parameters for four models for each simulated dataset, assuming exchangeable and block-exchangeable correlation structures, and both categorical and linear time effects, and applying Satterthwaite and Kenward-Roger corrections where we have fewer than $50$ clusters. Specifically, this study aims to $(1)$ assess whether the observed Type I error and empirical power for estimating the treatment effect align with their theoretical counterparts for correctly specified models; and $(2)$ assess the impact on inference for the treatment effect if the intracluster correlation structure is misspecified through performance metrics including Type I error and confidence interval coverage.

In the Methods Section, we describe the mixed model and the two correlation structures used in our study, along with details of the simulation study. The simulation study results are presented in the Results Section, followed by an analysis of a real trial example in the Disinvestment Trial Example Section. The Discussion Section provides a discussion of the findings.

## Methods

### Basic staircase designs

Staircase designs feature overlapping treatment sequences that initially involve one or more control periods, followed by one or more intervention periods, with gaps at the beginning or end where no measurements are taken. One type of staircase design is the balanced staircase design in which each sequence has an equal number of control periods before the treatment switch and intervention periods after the treatment switch [6]. A simplified version of the balanced staircase design is a basic staircase design, where each sequence consists of just one control period followed by one intervention period. Designs $2$ and $3$ in Figure 1 illustrate design schematics for two basic staircase designs with different numbers of sequences. We will explore a set of different basic staircase designs, considering various combinations of $S$ and $K$, where $S$ represents the number of sequences and $K$ indicates the number of clusters per sequence.

### Statistical model for continuous outcomes

We consider the following mixed effects model for a repeated cross-sectional design with a continuous outcome represented by ${Y}_{skti}$, the observation from participant $i=1,...,m$, in cluster $k=1,...,K$ assigned to sequence $s=1,...,S$ in period $t=s,s+1$:

1 $${Y}_{skti}={\beta }_{t}+\theta {X}_{st}+{CP}_{skt}+{\varepsilon }_{skti}$$

$${{\varvec{C}}{\varvec{P}}}_{sk}={\left({CP}_{sks},{CP}_{sks+1}\right)}^{T}\sim {N}_{2}\left(0, {\tau }^{2}{\varvec{R}}\right),{\varvec{R}}=\begin{bmatrix}1 & r \\r & 1\end{bmatrix}$$

$${\varepsilon }_{skti}\sim N\left(0, {\sigma }_{\varepsilon }^{2}\right)$$

The term ${\beta }_{t}$ is a fixed time effect corresponding to period $t$ (an alternative parameterisation is a linear time effect of ${\beta }_{1}+t{\beta }_{2}$). The term ${X}_{st}$ is the intervention indicator variable for sequence $s$ in period $t$, and $\theta$ is the intervention effect of interest. The term ${CP}_{skt}$ denotes the random effect associated with cluster $k$ assigned to sequence $s$ in period $t$ and ${\tau }^{2}{\varvec{R}}$ is the $2\times2$ covariance matrix of the cluster-period random effects, ${{\varvec{C}}{\varvec{P}}}_{sk}$, over the two periods of measurement. The elements of ${\varvec{R}}$ are defined according to the correlation structure being employed. We consider two intracluster correlation structures: the exchangeable [1] and the block-exchangeable (or constant between-period) [17, 18] intracluster correlation structures. The within-period intracluster correlation ($ICC$) denoted by $corr\left({Y}_{skti},{Y}_{skt{i}{\prime}}\right)=\frac{{\tau }^{2}}{{\tau }^{2}+{\sigma }_{\varepsilon }^{2}}=\rho$ describes the correlation between the outcomes of participants within the same cluster and period. The block-exchangeable structure has the form of model $\left(1\right)$ and assumes that the correlation between the outcomes of two participants measured in distinct periods is less than for participants measured in the same period: $corr({Y}_{skti},{Y}_{skt{\prime}i{\prime}})=\frac{{\tau }^{2}}{{\tau }^{2}+{\sigma }_{\varepsilon }^{2}}r=\rho r$, where the parameter $r\le 1$, known as the cluster-autocorrelation (CAC), represents the proportionate reduction in correlation between two distinct periods. When $r=1$, model $\left(1\right)$ reduces to the exchangeable correlation structure and assumes that the observations from any pair of participants within a cluster are equally correlated: $corr({Y}_{skti},{Y}_{skti{\prime}})=corr({Y}_{skti},{Y}_{skt{\prime}i{\prime}})=\rho$.

Grantham et al. [6] obtained explicit expressions for the variance of the treatment effect estimator, $var\left(\widehat{\theta }\right)$, for basic staircase designs where $\widehat{\theta }$ is the generalised least squares treatment effect estimator. The expressions under assumptions of categorical and linear period effects are represented as:

2 $${var\left(\widehat{\theta }\right)}_{cat}=\frac{2a{\left(1-\psi \right)}^{2}}{K\left[S\left(1-\psi \right)-\sqrt{1-{\psi }^{2}}\frac{{\left(1+\sqrt{1-{\psi }^{2}}\right)}^{S}-{\psi }^{S}}{{\left(1+\sqrt{1-{\psi }^{2}}\right)}^{S}+{\psi }^{S}}\right]}$$

3 $${var\left(\widehat{\theta }\right)}_{lin}=\frac{2a\left[\left({S}^{2}+2\right)-\left({S}^{2}-4\right)\psi \right]}{KS\left({S}^{2}-1\right)}$$

where $a=\frac{1+\left(m-1\right)\rho }{m}$ represents the variance of a cluster-period mean, $\psi =\frac{m\rho r}{1+\left(m-1\right)\rho }$ is the correlation between cluster-period means within the same cluster, and $m$ is again the number of participants measured in each cluster-period. Derivations of these expressions can be found in the Supporting Information of [6]. The above variance expressions, along with a standard power formula, can be used to calculate the power of a trial for a given effect size. The asymptotic power [1] for conducting a two-tailed test of size $\alpha$ to detect an effect size of ${\theta }_{d}$ is then given by:

$$Power=\Phi \left(\frac{{\theta }_{d}}{\sqrt{var\left(\widehat{\theta }\right)}}-{z}_{1-\frac{\alpha }{2}}\right)$$

where $\Phi$ is the cumulative standard Normal distribution, ${z}_{1-\frac{\alpha }{2}}$ is the $\left(1-\frac{\alpha }{2}\right)$ quantile of the standard Normal distribution, and $var\left(\widehat{\theta }\right)$ is the variance of the treatment effect estimator for the trial design of interest. When small-sample corrections are applied, the normal distribution is replaced by a t-distribution with degrees of freedom defined by the specific correction applied.

### Simulation study design

A simulation study was conducted to provide guidance on the design and analysis of basic staircase trials i.e., with one pre-switch control period and one post-switch intervention period. The details are presented below in accordance with the ADEMP (aims, data generating mechanisms, estimands, analysis methods, performance measures) framework for reporting simulation studies [36].

The specific aims of the study were:

1. To assess whether the observed Type I error and empirical power of Wald tests for testing the null hypothesis of no treatment effect align with their theoretical counterparts.
2. To assess Type I error rate, coverage, and bias of treatment effect estimates in the analysis of basic staircase designs when the correlation structure is misspecified in the analysis of data from the trial.

#### Data generating mechanism

We conducted a factorial simulation study, generating 1,000 datasets for each combination of parameters outlined in Table 1, resulting in a total of 576 configurations and $\text{576,000}$ datasets. With 1,000 replicates for each combination, the Monte Carlo standard error (MCSE) of a Type I error of 0.05 would be $0.0069$. We explored a variety of values for the design parameters: $S$ (total number of unique sequences), $K$ (total number of clusters assigned to each sequence), and $m$ (number of participants per cluster-period). We imposed a linear effect of time over the trial periods by setting ${\beta }_{t}=t$; this is consistent with both linear and categorical time effect parametrisations. We generated data under both exchangeable and block-exchangeable intracluster correlation structures, considering a range of intracluster correlation values, $\rho ,$ and, for the block-exchangeable structure, we also considered a range of cluster auto-correlation values $r$. Our selection of correlation parameter values was guided by estimates from the CLOUD bank repository [37], with a focus on within-period ICC values ranging from $0.01$ to $0.2$, and $r$ values between $0.5$ and $1$. The outcome for participant $i$ of cluster $k$ assigned to sequence $s$ in time $t$ was then randomly generated from the linear mixed model defined in Equation (1). Code to replicate our simulation study is available at https://github.com/EhsanRD/Staircase_simstudy.

**Table 1:** The parameters used when generating data for the simulation study

Parameter	Meaning	Values
$S$	Number of unique treatment sequences	$4$, $10$
$K$	Number of clusters randomised to each sequence	$1$, $5$, $10$
$m$	Number of participants per cluster-period	$10$, $50$, $100$
$\rho$	Intracluster correlation	$0.01$, $0.05$, $0.1$, $0.2$
$r$	Cluster autocorrelation	$1$, $0.95$, $0.8$, $0.5$
$\theta$	Treatment effect	$0$, $0.15$

#### Estimand

The primary focus is to test the null hypothesis that the intervention effect is zero (${H}_{0}: \theta =0$). Thus, the estimand of interest is the intervention effect, $\theta$.

#### Analysis methods

Four models were fit to each simulated dataset. That is, each simulated individual-level dataset was analysed using the mixed-effects model specified in Eq. (1), fitting models with both exchangeable and block-exchangeable correlation structures, and incorporating categorical and linear time effects in turn. Parameter estimation was carried out using REML. To address potential small-sample issues, the Satterthwaite (Sat) and the Kenward-Roger (KR) corrections were applied for all trial configurations except those where the total number of clusters exceeded $50$. Analyses were performed in R Studio $4.4.1$ with mixed models fit using the “lme4” package [38]. We used the “parameters” package in $R$ [39] to implement the Satterthwaite correction, and the “pbkrtest” package [40] to apply the Kenward-Roger correction. The “looplot” package was used to summarise the simulation results in nested loop plots [41].

The theoretical power for each scenario was calculated using the formulas provided above based on the asymptotic properties of the generalised least squares estimator.

#### Performance measures

For Aim $1$, we evaluated the bias in the estimation of the treatment effect, the Type I error and empirical power. Type I error was calculated as the proportion of times the null hypothesis was incorrectly rejected, i.e. concluding there is a non-null effect when there is actually a null treatment effect. Empirical power was calculated as the proportion of times the null hypothesis was correctly rejected. The MCSEs for each performance measure were calculated using standard methods [36]. For Aim $2$, we examined the impact of misspecifying the correlation structure on Type I error, 95% confidence interval coverage, and bias, again calculating MCSEs for each performance measure.

For both Aims 1 and 2, instances of non-convergence in the mixed models were flagged by warning messages from the “lmer” function. Singularity for the mixed effects model was characterised by an estimated variance of $0$ for the random cluster effect when an exchangeable model was fit, and by an estimated variance of $0$ for both the random cluster and the cluster-period effect when a block-exchangeable model was fit. We retained the estimates obtained from models with singular fits.

## Results

In the following two subsections we present the results for the two aims, for models with categorical period effects and the Satterthwaite correction method. Then in the subsequent subsection we describe results for additional analyses, such as using the Kenward-Roger adjustment, comparing coverage with and without small-sample corrections, and fitting models with a linear time effect.

No instances of model nonconvergence occurred over the entire simulation study. Figure S1 of Additional file 1 represents the rates of singularity for the mixed effect model fits for both exchangeable and block-exchangeable correlation structures. These rates were lower when the exchangeable correlation structure was assumed than when the block-exchangeable correlation structure was assumed across all scenarios. Rates of singularity for the exchangeable model fit were generally low, except in scenarios with a total of $4$ clusters or when the ICC was $0.01$. Singularity rates for the block-exchangeable model fit were high when the CAC = 1 (as is expected), when the total number of clusters was 4, and when the ICC was low (0.01), and decreased when there was greater decay (CAC $=0.5$) and the ICC increased. The singularity rates tended to decrease as the number of participants per cluster-period increased, but there were some instances where the rates were slightly higher for larger cluster-period sizes, most often in scenarios with a small number of clusters where the rates were all particularly high. The maximum singularity rate for the exchangeable model fit was $61.7\%$ for the scenario with an ICC of $0.01$, $4$ sequences, $1$ cluster per sequence, and a categorical time effect, with a CAC of $0.5$ and when there were $10$ participants per cluster-period. For the block-exchangeable model, the maximum singularity rate was $97.4\%$ for the same scenario but with a CAC of $0.95$, and when there were $50$ participants per cluster-period. We note that the singularity rate for the same configuration but with $10$ participants per cluster-period was similarly high: the MCSE bounds of $\pm 2\times$ MCSE for $50$ participants per cluster-period are $(96.4\%, 98.4\%)$ compared to $(95.0\%, 97.4\%)$ for the setting with $10$ participants per cluster-period. The slightly higher rate we observed for the larger cluster-period size is therefore likely driven by random simulation variation.

### Alignment of observed vs. theoretical Type I error and power for correctly specified models

In this section, we present the results only for scenarios where the model is correctly specified, i.e. when the analysis model matches the true data generating model. There was no bias in the estimation of the intervention effect (Figure S2 of Additional file 1); as was expected given the correct specification of the model for the mean outcome.

**Fig. 2:**
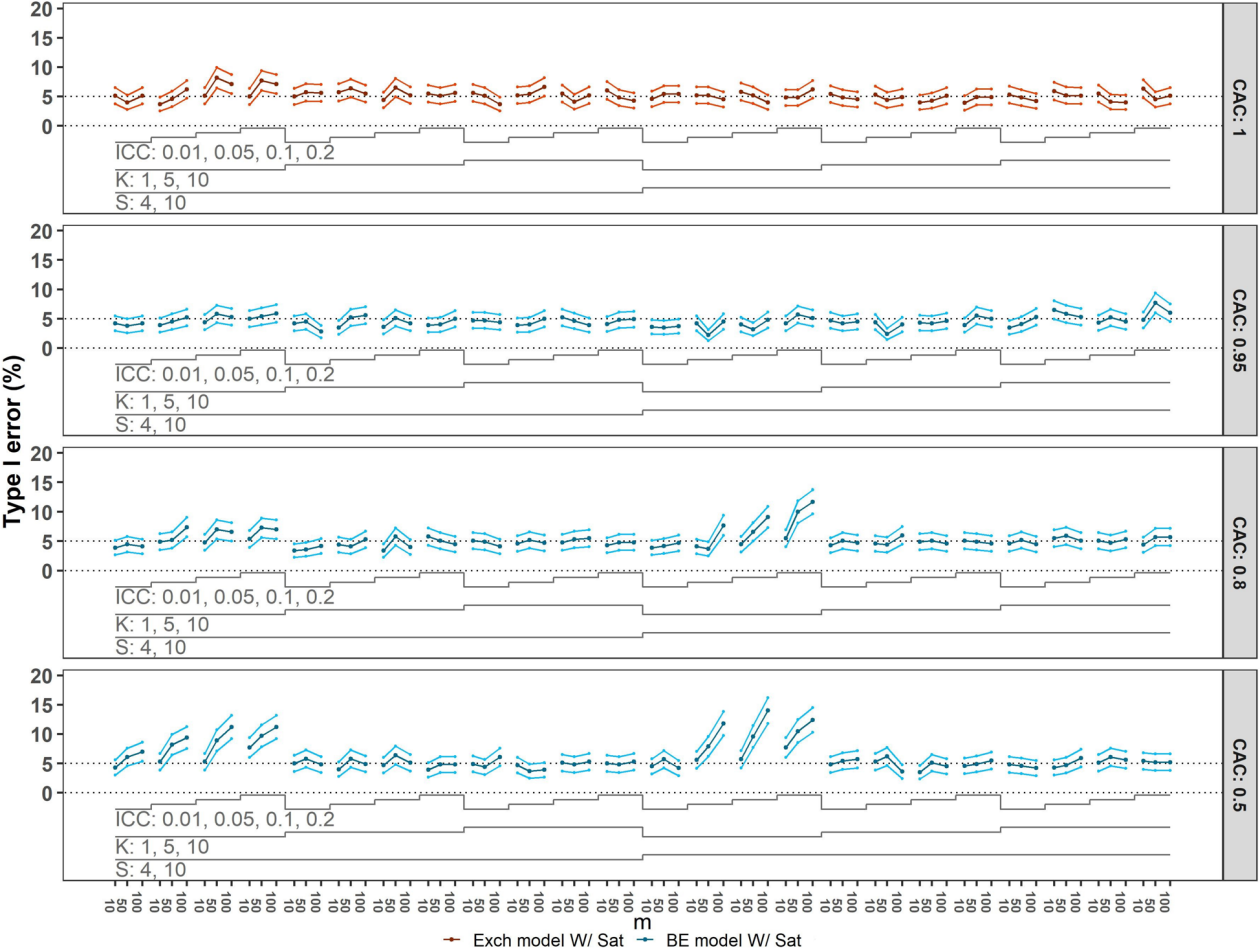
Type I error of correctly specified models with categorical time effects compared to the nominal $5\%$ level. Monte Carlo standard error (MCSE) bounds of $\pm 2\times$ MCSE are included. For configurations with $50$ or fewer clusters the Satterthwaite (Sat) small-sample correction method was used. Each panel corresponds to a CAC value: $1$ (top), $0.95$, $0.8$, $0.5$ (bottom). The panel includes the number of sequences $(S=4, 10)$, the number of clusters per sequence $(K=1, 5, 10)$, ICCs $(0.01, 0.05, 0.1, 0.2)$, and numbers of observations per cluster-period $(m=10, 50, 100)$, yielding a total of $288$ scenarios. BE = block-exchangeable; Exch = Exchangeable

Figure 2 displays the Type I error, our primary performance measure, for each scenario with $\theta$ $=0$. Each panel corresponds to a different CAC value for all combinations of ICC values, numbers of clusters per sequence, sequences, and participants per cluster-period. The top panel of the figure corresponds to the datasets generated under the exchangeable correlation structure, and all other panels correspond to datasets generated under the block-exchangeable structure with CAC $=0.95$ (second panel), CAC $=0.8$ (third panel) and CAC $=0.5$ (bottom panel).

The top panel of Figure 2 shows that when the true data generating model is the exchangeable model (i.e. CAC = $1$) and is analysed with an exchangeable model, the observed Type I error is generally well-controlled, achieving the nominal rate of $5\%$. However, there are a few scenarios where the Type I error exceeds the nominal rate of $5\%$. Specifically, this occurs in configurations with $4$ sequences, $1$ cluster per sequence, larger cluster-period sizes of $50$ or more, and an ICC of $0.1$ or higher. Considering now the datasets generated with a CAC ranging from $0.95$ to $0.5$ (the bottom three panels), and considering the fitting of block-exchangeable models (i.e. the correct model), the Type I error rate is inflated when there is one cluster $(K=1)$ per sequence (i.e. when there are a total of $4$ or $10$ clusters), with increasing Type I error rates observed as the ICC increases, as the values of the CAC decrease from $0.95$ to $0.5$, and as the number of participants per cluster-period increases.

**Fig. 3:**
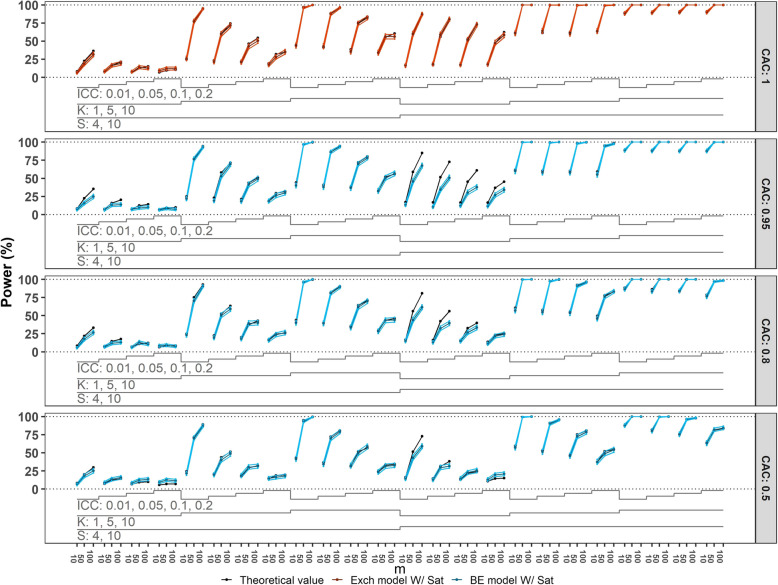
Power of correctly specified models with categorical time effects compared to the theoretical value. Monte Carlo standard error (MCSE) bounds of $\pm 2\times$ MCSE are included. For configurations with $50$ or fewer clusters the Satterthwaite (Sat) small-sample correction method was applied. Each panel corresponds to a CAC value: $1$ (top), $0.95$, $0.8$, $0.5$ (bottom). The panel includes the number of sequences $(S=4, 10)$, the number of clusters per sequence $(K=1, 5, 10)$, ICCs $(0.01, 0.05, 0.1, 0.2)$, and numbers of observations per cluster-period $(m=10, 50, 100)$, leading to a total of $288$ scenarios. BE = block-exchangeable; Exch = Exchangeable

Next, we compare the empirical power to the theoretical power, with results shown in Figure 3. Again, we focus on those situations where the correct model has been fit, but now to data generated with $\theta$ $=0.15$. In situations where the nominal Type I error is achieved, the empirical power for designs with $20$ clusters still shows some meaningful deviations from the theoretical value, with empirical power tending to be lower than the theoretical value when there is $1$ cluster per sequence. These deviations become less noticeable for designs with a total of $40$ or more clusters across all scenarios.

In scenarios where the Type I error is highly inflated (i.e. $4$ or $10$ clusters in total), the comparison between empirical power and theoretical power is not relevant, but we note that in these scenarios the empirical power is inflated.

### Impact of misspecification of correlation structure on inference for the treatment effect

We now present the results only for scenarios where the model fit to the trial data is incorrectly specified. In these scenarios, bias is minimal (Figure S3), as expected again given that the mean model is correctly specified.

**Fig. 4:**
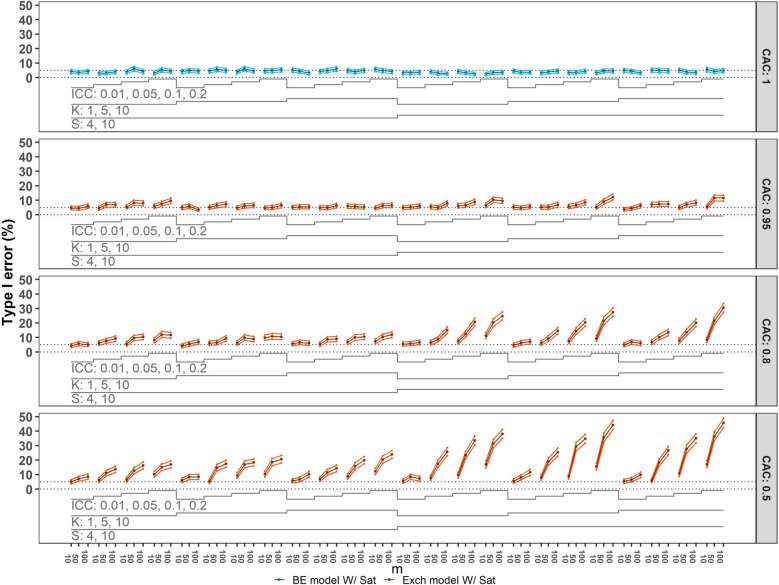
Type I error of overparameterised (top panel) and incorrectly specified models (all lower panels) with categorical time effects compared to the nominal $5\%$ value. Monte Carlo standard error (MCSE) bounds of $\pm 2\times$ MCSE are included. For configurations with $50$ or fewer clusters the Satterthwaite (Sat) small-sample correction method was used. Each panel corresponds to a CAC value: $1$ (top), $0.95$, $0.8$, $0.5$ (bottom). The panel includes the number of sequences $(S=4, 10)$, the number of clusters per sequence $(K=1, 5, 10)$, ICCs $(0.01, 0.05, 0.1, 0.2)$, and numbers of observations per cluster-period $(m=10, 50, 100)$, yielding a total of $288$ scenarios. BE = block-exchangeable; Exch = Exchangeable

**Fig. 5:**
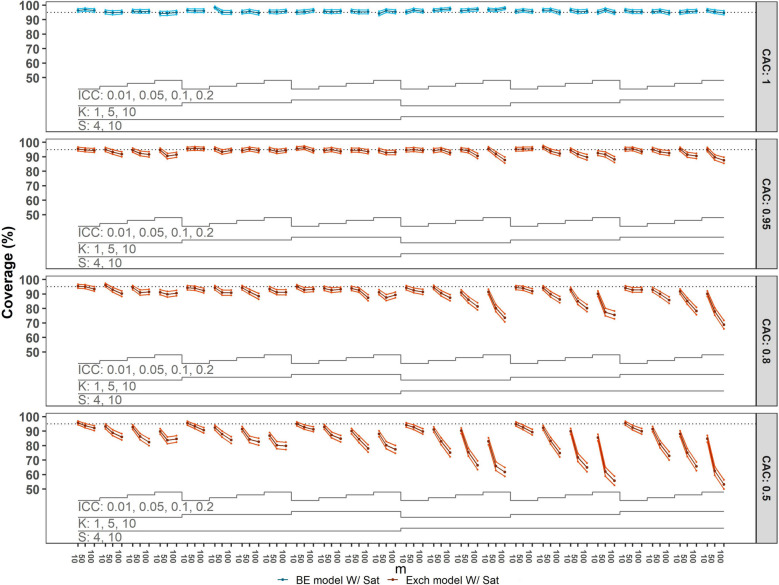
$95\%$ Confidence interval coverage of overparameterised (top) and incorrectly specified models (all lower panels) with categorical time effects compared to the target coverage of $0.95$ (dashed line). Monte Carlo standard error (MCSE) bounds of $\pm 2\times$ MCSE are included. For configurations with $50$ or fewer clusters the Satterthwaite (Sat) small-sample correction method was used. Each panel corresponds to a CAC value: $1$ (top), $0.95$, $0.8$, $0.5$ (bottom). The panel includes the number of sequences $(S=4, 10)$, the number of clusters per sequence $(K=1, 5, 10)$, ICCs $(0.01, 0.05, 0.1, 0.2)$, and numbers of observations per cluster-period $(m=10, 50, 100)$, yielding a total of $288$ scenarios. BE = block-exchangeable; Exch = Exchangeable

The bottom three panels of Figure 4 indicate that if the true underlying model is block-exchangeable but an exchangeable model is fit, resulting in a misspecified correlation structure, the empirical Type I error is excessively high. This becomes more pronounced with more decay as the values of CAC decrease from $0.95$ to $0.5$, and both ICC and the number of participants per cluster-period increase. Similarly, coverage is notably too low, as illustrated in Figure 5, which displays the confidence interval coverage results from the analysis of datasets generated with $\theta$= 0.15, consistent with observations of inflated Type I error rates. For these scenarios (bottom three panels), the impact of misspecification of the correlation structure is more severe when there is more decay. Furthermore, coverage tends to decrease with an increase in the number of clusters, participants per cluster-period, and larger ICC values.

When fitting the block-exchangeable model while the true structure is the exchangeable model, the model is over-parameterised rather than misspecified. When the CAC is $1$ and the block-exchangeable model is fit (Figure 4, top panel), the observed Type I error is slightly lower than the nominal value when there is only 1 cluster per sequence (i.e. $4$ or $10$ clusters in total). As shown in Figure 5 (top panel), confidence interval coverage is slightly higher than the nominal 95% level for these scenarios where the over-parameterised model is fit, in accordance with the Type I error pattern observed in Figure 4.

### Additional results

In Section C of Additional file 1, we provide the results of applying the Kenward-Roger correction on Type I error, power, and coverage (Figures S4-S7). These results are similar across all configurations to those obtained when applying the Satterthwaite small-sample correctionin Sections, with the exception of the block-exchangeable fit for designs with $10$ or fewer clusters. Specifically, for these designs with only one cluster per sequence, the Kenward-Roger correction is very conservative, resulting in Type I error rates virtually equal to 0. Consequently, both the power and coverage performance measures are inaccurate, with power being too low and coverage being overestimated.

In Section $D$, we compare the performance of the Satterthwaite and Kenward-Roger small-sample corrections to models fit without these corrections. The results in Figures S8 and S10 indicate that when fitting an exchangeable model with a very small number of clusters ($4$ in total), applying either the Satterthwaite or Kenward-Roger approximation enhances coverage accuracy, although this correction does not fully correct the coverage to the nominal 95% level. When fitting a block-exchangeable model, the Satterthwaite correction improves coverage accuracy for designs with 1 cluster per sequence ($4$ or $10$ clusters in total) (Figures S9 and S11). For these configurations, as the CAC increases, the gap between corrected and uncorrected coverage becomes more evident.

In Section $E$, results are provided when models with a linear time effect as described in Section are fitted. Across all scenarios, the results are similar to those obtained when categorical time effects are fitted except in those scenarios with $1$ cluster per sequence. In these scenarios, there is some improvement in performance measures when using the Kenward-Roger correction compared to results for models with categorical time effects, with Type I error and power aligning more closely with their theoretical values, although they still remain below acceptable levels. For these configurations, the observed Type I error with the Kenward-Roger correction is still too conservative, but not as conservative as with the categorical time effect. Our key findings are summarised in Table 2.

**Table 2:** Key simulation findings

**Type I error and power for correctly specified models**
Observed Type I error and empirical power are well aligned with the theoretical values if the intracluster correlation structure is correctly specified and the design includes $40$ clusters or more. One cluster per sequence leads to inflated Type I error, even when the Satterthwaite correction is applied. Furthermore, when applying the Kenward-Roger correction to the block-exchangeable model, the Type I error becomes too conservative if there is only one cluster per sequence
**Type I error and coverage when fitting a misspecified model**
Misspecification of the intracluster correlation structure (i.e. fitting models with an exchangeable structure when the block-exchangeable structure was used to generate the data) leads to inflated Type I error, and confidence interval coverage does not reach the nominal 95% level
**Linear time effect vs categorical time effects**
When the assumptions for a linear time effect are satisfied, improved performance measures can be observed for designs with 1 cluster per sequence. For larger numbers of clusters, there is minimal difference between performance measures when categorical or linear time effects are assumed

## Disinvestment trial example

To illustrate the impacts of different modelling choices as explored in our simulation study on inference about the treatment effect in a real-world staircase setting, we now consider a trial that was conducted in Australia aiming to evaluate the impact of disinvesting from and later reinvesting in weekend allied health services at two hospitals [42]. In this study, the researchers conducted two stepped wedge trials in $12$ acute medical and surgical wards across Dandenong Hospital and Footscray Hospital, taking place over different but overlapping time frames. For this example, we use data from the reinvestment phase at Dandenong Hospital, which is publicly accessible online [42].

**Fig. 6:**
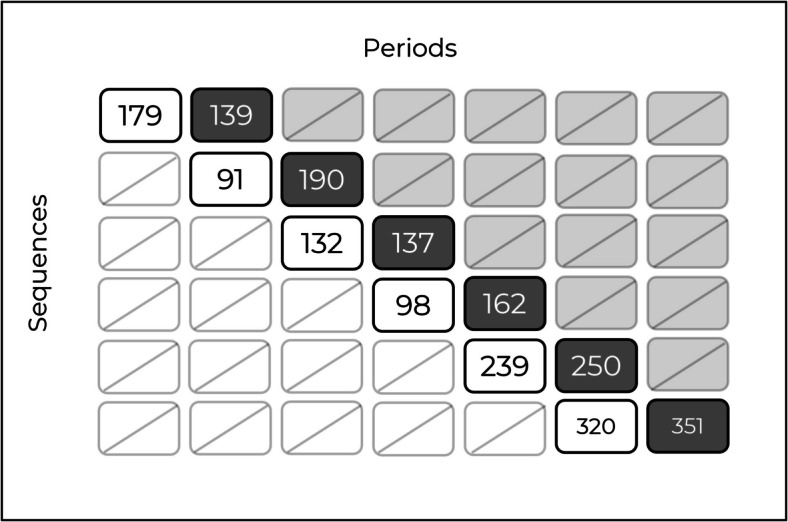
The embedded basic staircase design from the Disinvestment stepped wedge trial example, displaying the number of participants per cluster-period cell. Cluster-period cells with gray shading indicate the presence of new weekend allied health services, cells with white shading represent those without, and cells with a slash indicate cluster-periods where no measurements are taken

The reinvestment phase of the Disinvestment trial involved a $6$-sequence stepped wedge design spanning $7$ distinct time periods, where each ward, treated as a cluster, was randomly assigned to a treatment sequence; one ward from each hospital was randomised to each sequence. In this trial, a new weekend allied health service was incrementally introduced across the wards after an old service had been removed. We consider the log-transformed length of stay for patients, measured in days, as the relevant outcome. In the original stepped wedge trial, there were a total of $\text{8,444}$ observations, with an average of $201$ patients per cluster-period. We extracted a subset of the data that corresponds to the time periods of measurement from an embedded basic staircase design. After deriving a staircase design from this trial, there were $\text{2,281}$ total observations, with an average of $190$ patients per cluster-period. The embedded basic staircase design with the number of participants in each cluster-period is shown in Figure 6. Cluster-period cells with gray shading indicate the presence of new weekend allied health services, cells with white shading represent those without, and cells with a slash indicate cluster-periods where no measurements are taken.

We fit the four models that incorporate each combination of exchangeable and block-exchangeable correlation structure and categorical and linear time effects to this dataset. We also apply Kenward-Roger and Satterthwaite corrections to each fitted model. Results are presented in Table 3 and include the treatment effect estimates, their standard errors and confidence intervals, and the estimated degrees of freedom when a correction is applied. Additionally, we report the correlation parameter estimates.

**Table 3:** Inference for the treatment effect and correlation parameter estimates for the embedded basic staircase design with $S=6$, $K=1$, $\overline{m }=190$ for the Disinvestment trial example. $\widehat{\rho }$ and $\widehat{r}$ are the estimates of ICC and CAC, respectively. $\widehat{\theta }$, $se(\widehat{\theta })$, and $CI(\widehat{\theta })$ are the estimate of the treatment effect, the standard error of the treatment effect, and $95\%$ confidence interval of the treatment effect respectively

Model fit	Time period	ICC	CAC	Treatment effect	No correction		Satterthwaite correction			Kenward-Roger correction		
		$\boldsymbol{\widehat{\rho }}$	$\boldsymbol{\widehat{r}}$	${\boldsymbol{\widehat{\theta }}}$	$\boldsymbol{se\left(\widehat{\theta }\right)}$	95% $\boldsymbol{CI\left(\widehat{\theta }\right)}$	$\boldsymbol{se\left(\widehat{\theta }\right)}$	***DoF***	95% $\boldsymbol{CI\left(\widehat{\theta }\right)}$	$\boldsymbol{se\left(\widehat{\theta }\right)}$	***DoF***	95% $\boldsymbol{CI\left(\widehat{\theta }\right)}$
Exchangeable	Categorical	0.05	-	1.34	0.37	(0.60, 2.07)	0.37	12.9	(0.53, 2.15)	0.38	12.24	(0.52, 2.15)
Block-exchangeable	Categorical	0.05	1^a^	1.34	0.37	(0.60, 2.07)	0.37	12.9	(0.53, 2.15)	0.50	0.1	(−2.7 × 10^12^, 2.7 × 10^12^)
Exchangeable	Linear	0.04	-	1.23	0.34	(0.57, 1.88)	0.34	10.72	(0.49, 1.97)	0.34	10.94	(0.49, 1.97)
Block-exchangeable	Linear	0.04	0.95	0.36	0.36	(0.55, 1.98)	0.36	8.48	(0.44, 2.09)	0.37	8.26	(0.42, 2.11)

^a^Estimates were obtained from a singular fit, with the variance of the cluster-period random effect equal to zero

The majority of the confidence intervals for the treatment effect obtained from these methods suggest a non-null treatment effect, indicating that the log-length of stay increases by around 1.3 units, with confidence intervals of around $0.5$ to $2$, when the service is re-implemented (excluding the Kenward-Roger confidence interval obtained when categorical time effects are included, which we discuss further below). This trial features $6$ sequences, with each cluster period cell containing over $100$ observations. Out of all the designs considered in the simulation study (Table 1), the one that most closely resembles this trial has $4$ sequences, with $1$ cluster per sequence and $100$ observations per cluster period. Based on the parameter estimates, $\widehat{\rho }$ and $\widehat{r}$ from Table 3, the most relevant correlation parameters are an ICC of $\rho =0.05$ and CAC of $r=0.95$. Our simulation study results indicate that, in these scenarios, the empirical Type I error rate from a block-exchangeable model fit with a categorical time effect and Kenward-Roger correction fails to align with the nominal Type I error rate, producing inaccurate and overly conservative results (Figure S4). In light of these results, is it unsurprising that inference for the treatment effect for the Disinvestment trial when using the Kenward-Roger correction is overly conservative, with an extremely low degrees of freedom of $0.1$ and correspondingly exceptionally wide confidence intervals for the treatment effect of $(-2.7\times {10}^{12},$ $2.7\times {10}^{12})$ (Table 3). These results underscore the inadequacy and inappropriateness of the estimates obtained from this approach.

Based on our findings from the simulation study, when a linear time effect is valid and the true model is block-exchangeable with CAC of $0.95$, fitting an exchangeable model (misspecifying the model) with either the Satterthwaite or Kenward-Roger correction still yields acceptable confidence interval coverage for the treatment effect (Figures S14 and S18). Similar conclusions can be reached when fitting a block-exchangeable model while the true model is exchangeable (i.e., overparameterising the model). As shown in Table 3, the confidence intervals for the treatment effect are similar for the block-exchangeable and exchangeable models where a linear time effect was assumed. To be more precise, our findings from the simulation study are consistent with the results presented in Table 3 from the trial analysis for this scenario when the time effect is linear: (i) the block-exchangeable model yields slightly more conservative results than the exchangeable model, though the difference between them is minimal. In Table 3, it can be observed that the block-exchangeable model results in slightly wider confidence intervals than the exchangeable model. (ii) The block-exchangeable model, when using the Kenward-Roger correction, produces somewhat more conservative results than when using the Satterthwaite correction; however, in contrast to the confidence interval obtained when categorical period effects were included, the confidence interval obtained via the Kenward-Roger correction includes a reasonable range of values. (iii) The exchangeable model, regardless of whether the Kenward-Roger or Satterthwaite correction is applied, produces similar results.

## Discussion

In this paper we investigated the alignment between observed and theoretical Type I error and power for correctly specified models when analysing data from basic staircase designs via linear mixed models, as well as the impact of misspecifying the correlation structure on treatment effect inference. The findings reveal that with categorical period effects and a Satterthwaite small-sample correction, the empirical power closely aligns with the theoretical power values when the correlation structure is correctly specified and the design includes a sufficient number of clusters, at least $40$ clusters in total. Notably, for designs with $1$ cluster per sequence, Type I error is inflated. Scenarios where the correlation structure is misspecified, i.e. fitting an exchangeable model while the true correlation structure is block-exchangeable, often demonstrated poor performance (including under-coverage and inflated Type I error rates), particularly when there was more decay in correlation. This indicates that the results can be misleading and unreliable, as the 95% confidence interval coverage for the treatment effect can be too narrow and fail to include the true value as often as expected. Results where the Kenward-Roger small-sample correction method is applied are similar to those where the Satterthwaite correction is applied, except in cases where a block-exchangeable model is used for designs with $1$ cluster per sequence, where the difference is more noticeable. In these cases, particularly when the model includes categorical time effects, the Kenward-Roger small-sample correction is far too conservative, yielding low empirical Type I error near zero. As illustrated in Section $B$ in Additional file 1, for any CAC value, the bias is negligible when fitting either the exchangeable or block-exchangeable models, indicating that the treatment effect estimates are unbiased, as expected. Additionally, when a linear time effect is assumed, the results are similar to those obtained when categorical time effects are included, with only slight variations observed in some scenarios. With a linear time effect, the observed Type I error improves and aligns more closely with the nominal level for these configurations compared to categorical time effect. These results are detailed in Section $E$ of Additional file 1.

The small-sample corrections applied in this work aim to improve control of the Type I error rate when few clusters are included. These corrections involve approximating the distribution of test statistics with a t-distribution, and estimating the degrees of freedom; the Kenward-Roger correction also adjusts standard errors. These corrections can be based on either the observed information matrix (OIM) or expected information matrix (EIM). Different statistical software offers different variations of these corrections. For instance, in Stata, users can choose between OIM and EIM when applying either the Kenward-Roger and Satterthwaite corrections. In R, we were unable to select the information matrix variant for each correction, and were unable to apply both the Kenward-Roger and Satterthwaite small-sample corrections from one single package in R. While both corrections are provided in the ‘parameters’ and ‘pbkrtest’ packages, we opted to use the Kenward-Roger correction from the ‘pbkrtest’ package, which is based on the EIM, as we found the use of the correction in the ‘parameters’ package to be highly time-consuming. Both packages appear to produce the same results for the Kenward-Roger correction, as they both rely on the EIM. On the other hand, for the Satterthwaite correction, we chose the ‘parameters’ packages which uses the OIM, since we were unable to obtain estimates from the ‘pbkrtest’ package using this correction. Further investigation into the differences between EIM and OIM in the analysis of staircase and other trial designs with a small number of clusters is warranted.

The number of clusters has an important effect on Type I error rates in analysing basic staircase designs using linear mixed effect models. Our findings suggest that staircase designs with one pre-intervention period and one post-intervention period require at least $40$ clusters in total to yield reliable results under correctly specified models. However, when a decay in the intracluster correlation from one period to the next is ignored, even with a large number of clusters, an inflated Type I error rate, and as a result incorrect inference, can occur. The effect of incorrectly ignoring the decay in the correlation becomes more severe when there is more decay (i.e. small cluster autocorrelation). At the analysis stage, one approach is to fit a decay model and obtain the CAC estimate; if there is likely to be substantial decay, fitting a block-exchangeable model to the trial data is strongly recommended. Given the potentially large impact of ignoring a decay, it is highly recommended to incorporate a correlation structure that accounts for decay at the design stage of staircase trials in the sample size calculations to ensure studies are adequately powered to detect treatment effects of interest. If the assumption of a linear effect of time on the outcome is likely to be valid, specifying a linear form for time in the model can offer benefits, such as lower inflation of the Type I error rate and higher confidence interval coverage for the treatment effect. This is most relevant for designs with a small number of clusters (e.g., $10$ clusters), where specifying a linear time effect generally leads to similar or slightly better performance than using categorical time effects, particularly if there is no decay in the intracluster correlation structure.

Although our findings favour the Satterthwaite correction over the Kenward-Roger correction for trials with only $1$ cluster per sequence or a total of $10$ clusters or fewer, there is still a risk of Type I error rate inflation. There have been several studies evaluating the performance of data analysis methods of data from stepped wedge designs, where the analysis model utilised the same intracluster correlation structure employed in generating the data [25, 31, 43, 44]. Grantham et al. [31] found that using REML with the Kenward-Roger correction performed well in analysing stepped wedge cluster randomised trials using block-exchangeable correlation structure even with a limited number of clusters. This contrasts with our findings for basic staircase designs: we found that block-exchangeable models and Kenward-Roger correction produced inaccurate results for the designs with $1$ cluster per sequence. Another simulation study by Lee et al. [43] in the context of stepped wedge designs demonstrated that the exchangeable model is susceptible to inadequate coverage and inflated Type I error rates, and that applying Satterthwaite or Kenward–Roger small-sample corrections could result in inflated or overly conservative type I errors, respectively. These findings are consistent with ours for staircase designs.

Our findings regarding the impact of misspecifying the intracluster correlation structure for basic staircase designs are broadly consistent with other studies considering the impact of similar forms of misspecification of the correlation structure for complete stepped wedge designs. Our results are consistent with the analytical findings of Kasza et al. [20], which show that misspecifying the intracluster correlation structure in the analysis of data from a stepped wedge trial can lead to incorrect conclusions regarding the treatment effect. They showed that the confidence interval for the treatment effect becomes too narrow when a simpler model is incorrectly assumed instead of a more complex model, consistent with the very low coverage observed in our study for scenarios with exchangeable model fit when there is a decay. Ouyang et al. [21] explored the use of robust variance estimators to address the challenges of random-effects misspecification in the analysis of data from stepped wedge designs when more complex correlation structures in linear mixed models are computationally difficult to specify for stepped wedge trials, via a simulation study. They also showed that incorporating a robust variance estimator can enhance the validity of statistical inference for a misspecified linear mixed model; further work is required to assess the impacts of this work for staircase designs.

While our simulation study explored a wide variety of scenarios within the context of basic staircase designs, certain limitations exist. We concluded that a minimum of $40$ clusters ($10$ sequences with $4$ clusters per sequence) is necessary to achieve acceptable performance measures under correctly specified models without any corrections for basic staircase designs. This work can be extended to incorporate other staircase design variants beyond the basic staircase such as those with more than two measurement periods in each sequence. More complex correlation structures such as discrete-time decay correlation would then be applicable, and associated impacts of correlation structure misspecification would need to be explored [6]. We focused on linear mixed models with normally distributed errors in basic staircase designs involving repeated cross-sectional measurements and continuous outcomes. Our work could be extended to closed or open cohort designs by incorporating participant-level random effects in Model $(1)$. Binary outcomes are commonly of interest in longitudinal cluster trials [45], and the simulation study framework presented in this study can be adapted for implementation within generalised linear mixed models or fitting models via generalised estimating equations.

## Conclusion

Staircase designs with only one cluster per sequence are not recommended due to their poor statistical properties and should be used with caution. The Satterthwaite correction is recommended for the analysis of data via linear mixed models from staircase designs with fewer than $40$ clusters. Additionally, using a correlation structure that allows for decay is preferable for making valid inferences for the estimation of the treatment effect when analysing data from basic staircase designs.

## Supplementary Information

Additional file 1.

## Data Availability

Code for running the simulation study and for performing the analysis of the Disinvestment data is available at https://github.com/EhsanRD/Staircase_simstudy. The disinvestment data are available at https://journals.plos.org/plosmedicine/article?id = 10.1371/journal.pmed.1002412.

## Abbreviations

ICC: Intracluster correlation
ML: Maximum likelihood
REML: Restricted maximum likelihood
CAC: Cluster-autocorrelation
Sat: Satterthwaite
KR: Kenward-Roger
MCSE: Monte Carlo standard error
Exch: Exchangeable
BE: Block-exchangeable
SE: Standard error
