# Supplementary material for: Inference for the treatment effect in staircase designs with continuous outcomes: a simulation study
Source: BMC Med Res Methodol. 2025 May 10;25:127. doi: 10.1186/s12874-025-02567-5 (PMC12065208; doi:10.1186/s12874-025-02567-5)
Supplement: Supplementary file 1 — Additional file 1. [file 12874_2025_2567_MOESM1_ESM.pdf]

Additional file 1: Inference for the treatment effect in staircase designs with continuous outcomes: a simulation study

Ehsan Rezaei-Darzi, Kelsey L Grantham, Andrew B Forbes, Jessica Kasza

School of Public Health and Preventive Medicine, Monash University, Australia

A Singular model fits

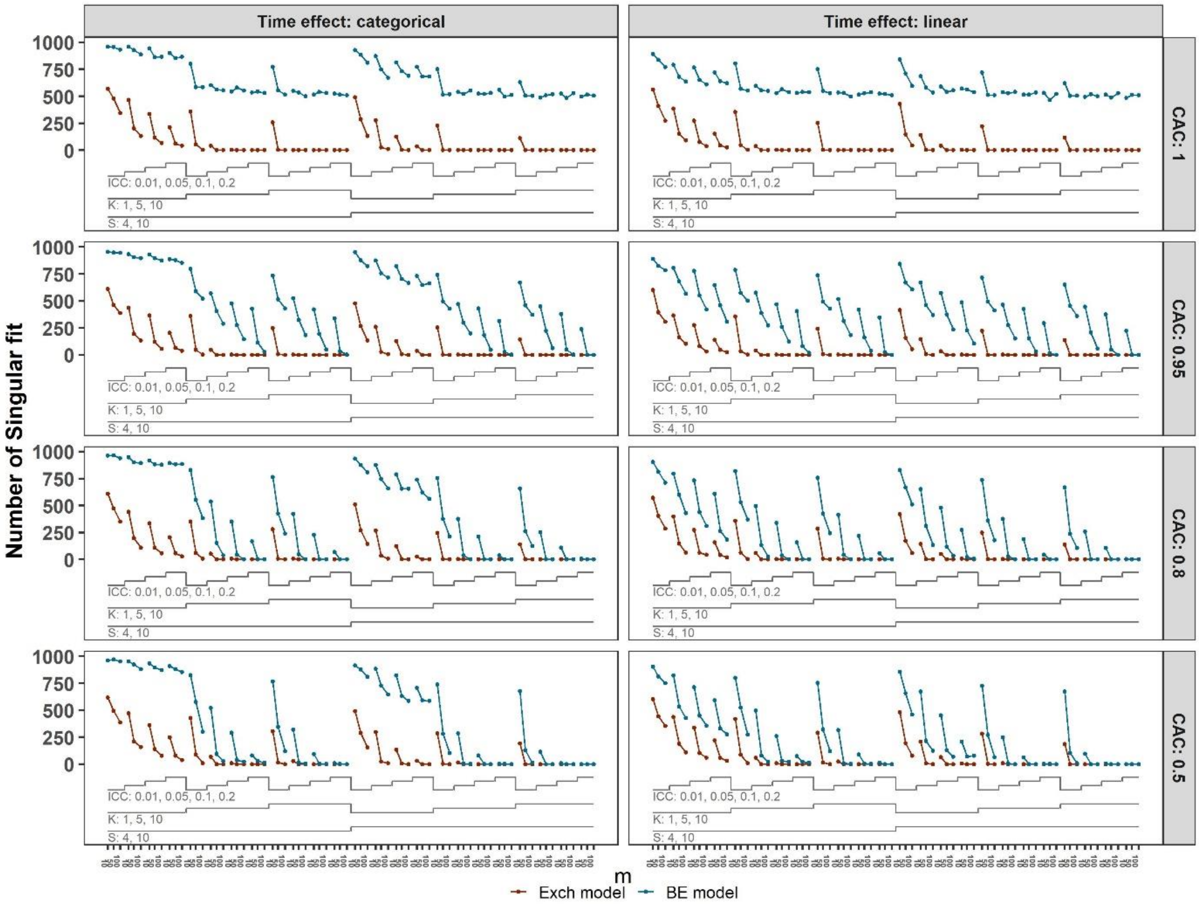

Figure $S1$. Singularity rates for the mixed effect model fits for both exchangeable (Exch) and block-exchangeable (BE) correlation structures. Each panel corresponds to a CAC value: 1 (top), 0.95, 0.8, 0.5 (bottom) and time effect: categorical or linear. The panel includes the number of sequences ($S = 4, 10$), the number of clusters per sequence ($K = 1, 5, 10$), ICCs ($0.01, 0.05, 0.1, 0.2$), and numbers of observations per cluster-period ($m = 10, 50, 100$), yielding a total of 288 scenarios.

B Bias for treatment effect

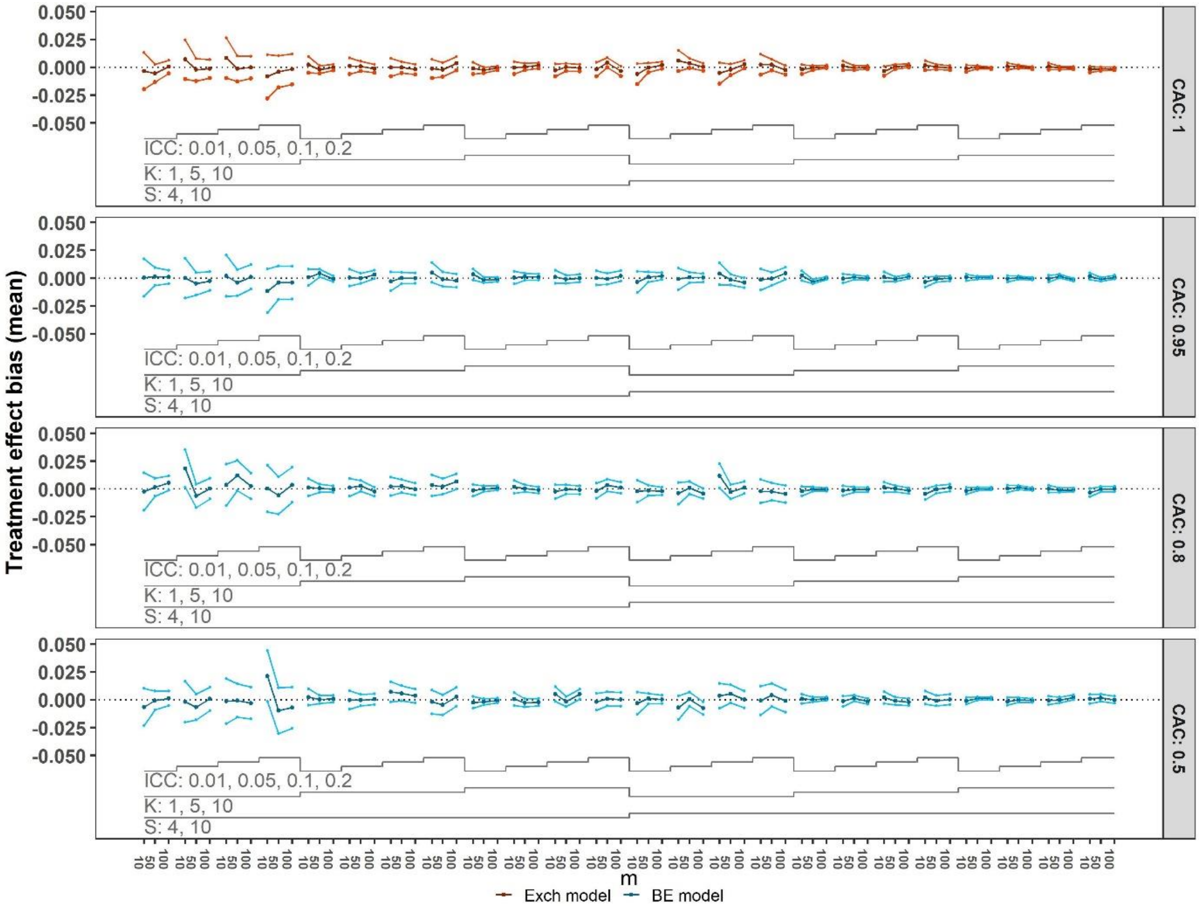

Figure $S2$. Bias for treatment effect of correctly specified models with categorical time effects. Monte Carlo standard error (MCSE) bounds of $\pm\, 2 \times$ MCSE are included. Each panel corresponds to a CAC value: 1 (top), 0.95, 0.8, 0.5 (bottom). The panel includes the number of sequences ($S = 4, 10$), the number of clusters per sequence ($K = 1, 5, 10$), ICCs ($0.01, 0.05, 0.1, 0.2$), and numbers of observations per cluster-period ($m = 10, 50, 100$), yielding a total of 288 scenarios. BE=block-exchangeable; Exch=Exchangeable.

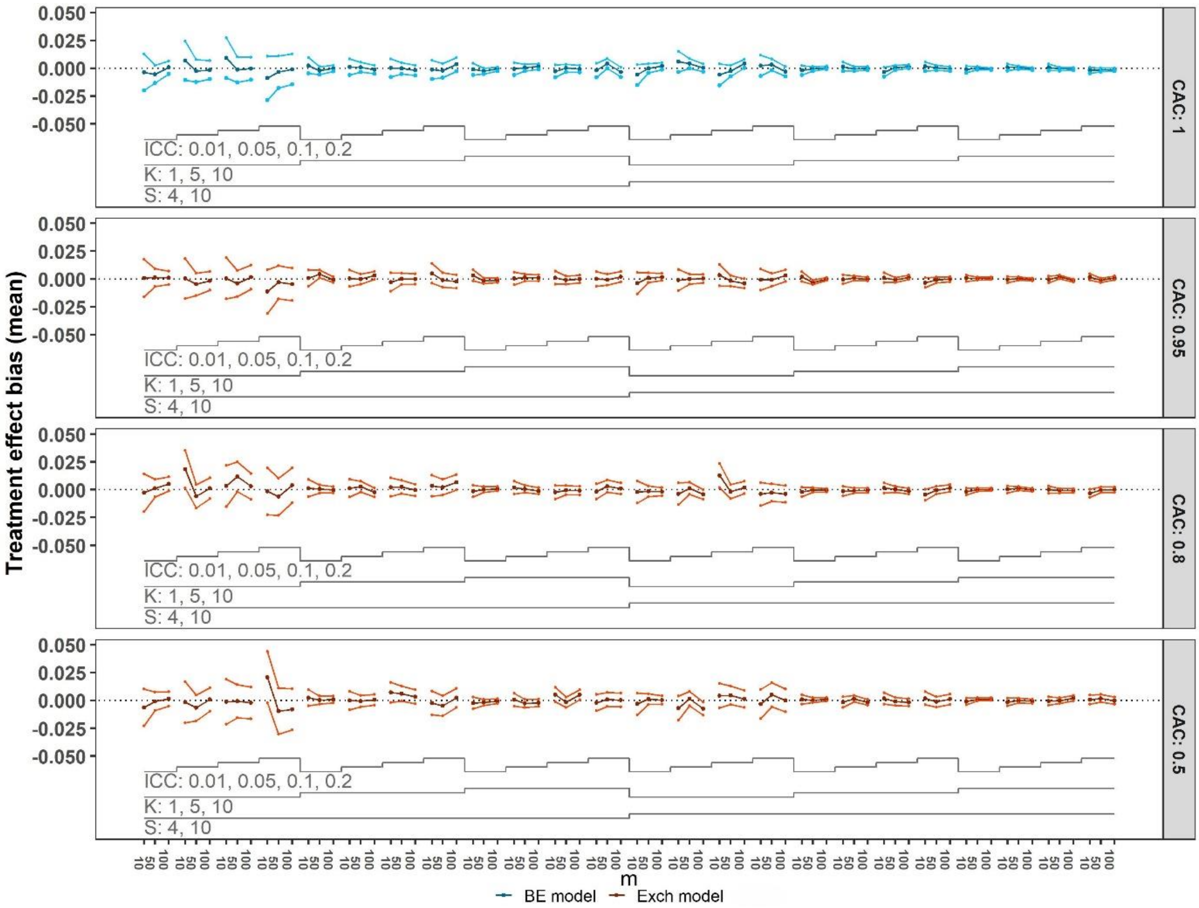

Figure $S3$. Bias for treatment effect of overparameterised (top panel) and incorrectly specified models (all lower panels) with categorical time effects. Monte Carlo standard error (MCSE) bounds of $\pm 2 \times$ MCSE are included. Each panel corresponds to a CAC value: 1 (top), 0.95, 0.8, 0.5 (bottom). The panel includes the number of sequences ($S = 4, 10$), the number of clusters per sequence ($K = 1, 5, 10$), ICCs ($0.01, 0.05, 0.1, 0.2$), and numbers of observations per cluster-period ($m = 10, 50, 100$), yielding a total of 288 scenarios. BE=block-exchangeable; Exch=Exchangeable.

C Performance measures using Kenward-Roger small-sample correction

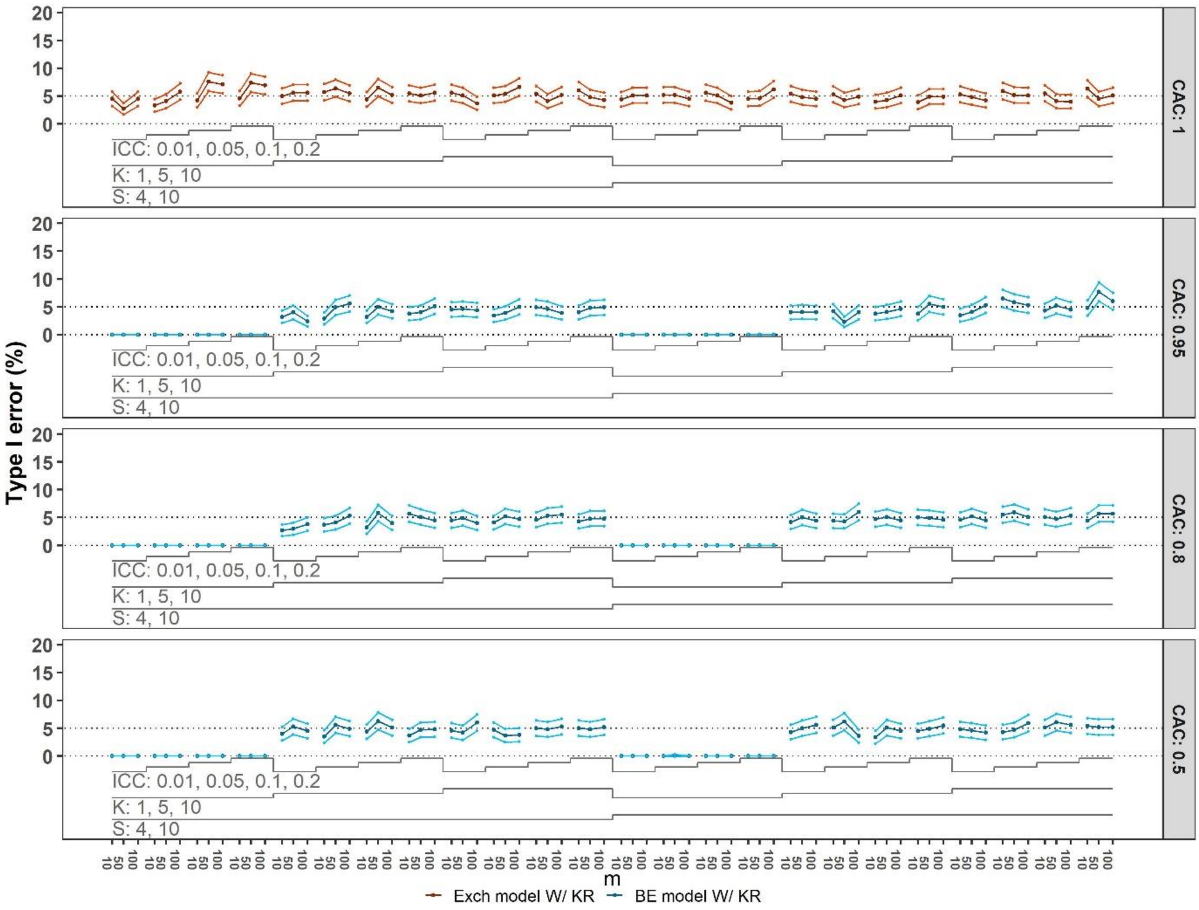

Figure $S4$. Type I error of correctly specified models with categorical time effects compared to the nominal 5% level. Monte Carlo standard error (MCSE) bounds of $\pm 2 \times$ MCSE are included. For configurations with 50 or fewer clusters the Kenward-Roger (KR) small-sample correction method was used. Each panel corresponds to a CAC value: 1 (top), 0.95, 0.8, 0.5 (bottom). The panel includes the number of sequences ($S = 4, 10$), the number of clusters per sequence ($K = 1, 5, 10$), ICCs ($0.01, 0.05, 0.1, 0.2$), and numbers of observations per cluster-period ($m = 10, 50, 100$), yielding a total of 288 scenarios. BE=block-exchangeable; Exch=Exchangeable.

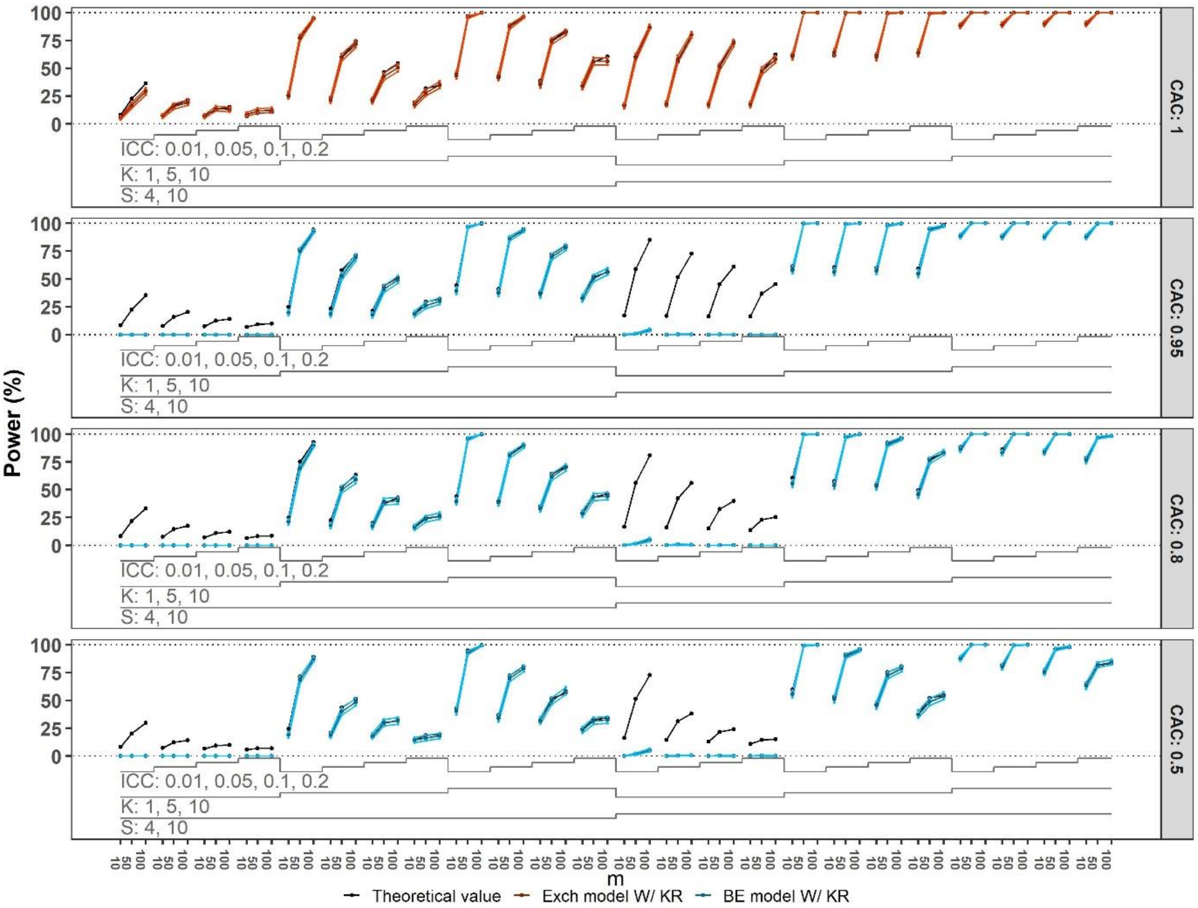

Figure $S5$. Power of correctly specified models with categorical time effects compared to the theoretical value. Monte Carlo standard error (MCSE) bounds of $\pm 2 \times$ MCSE are included. For configurations with 50 or fewer clusters the Kenward-Roger (KR) small-sample correction method was used. Each panel corresponds to a CAC value: 1 (top), 0.95, 0.8, 0.5 (bottom). The panel includes the number of sequences ($S = 4, 10$), the number of clusters per sequence ($K = 1, 5, 10$), ICCs ($0.01, 0.05, 0.1, 0.2$), and numbers of observations per cluster-period ($m = 10, 50, 100$), yielding a total of 288 scenarios. BE=block-exchangeable; Exch=Exchangeable.

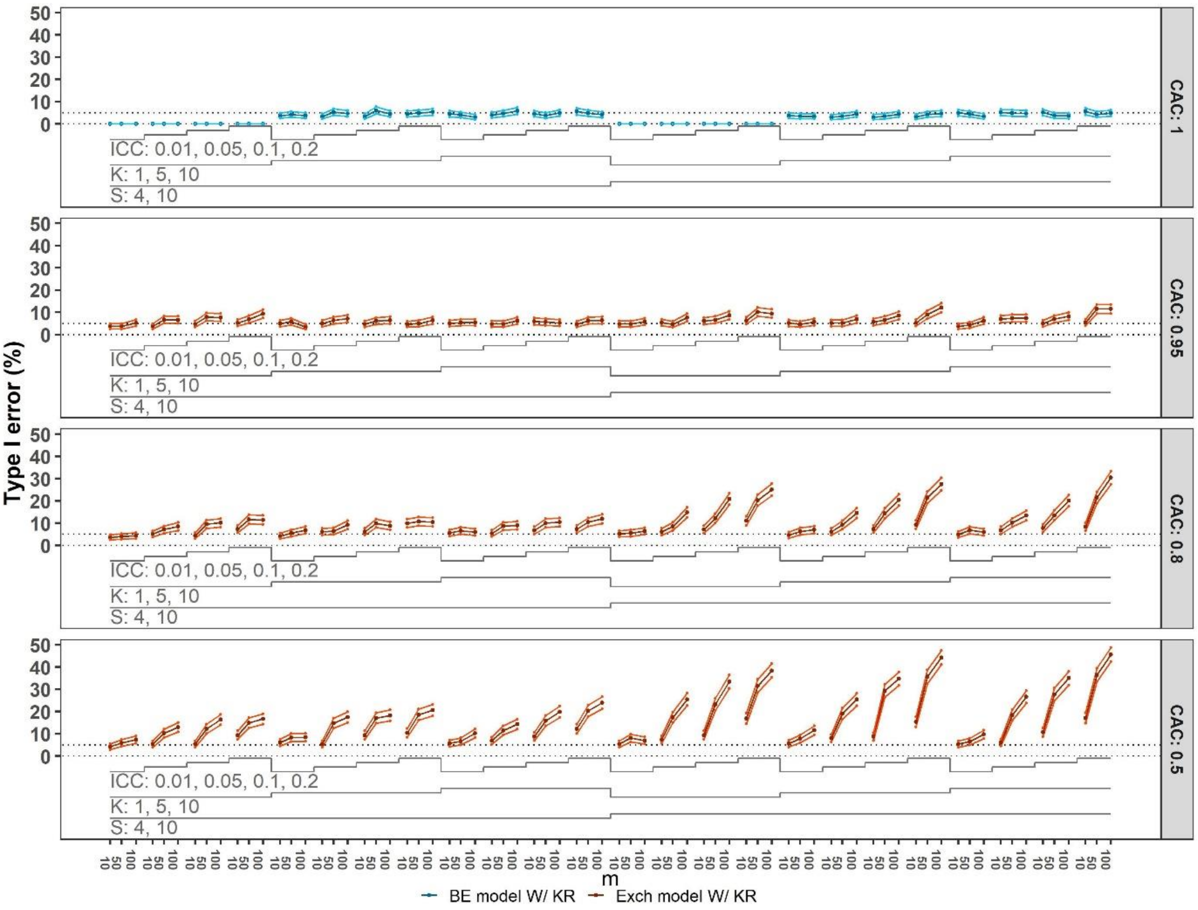

Figure $S6$. Type I error of overparameterised (top panel) and incorrectly specified models (all lower panels) with categorical time effects compared to the nominal 5% value. Monte Carlo standard error (MCSE) bounds of $\pm\ 2 \times$ MCSE are included. For configurations with 50 or fewer clusters the Kenward-Roger (KR) small-sample correction method was used. Each panel corresponds to a CAC value: 1 (top), 0.95, 0.8, 0.5 (bottom). The panel includes the number of sequences ($S = 4, 10$), the number of clusters per sequence ($K = 1, 5, 10$), ICCs ($0.01, 0.05, 0.1, 0.2$), and numbers of observations per cluster-period ($m = 10, 50, 100$), yielding a total of 288 scenarios. BE=block-exchangeable; Exch=Exchangeable.

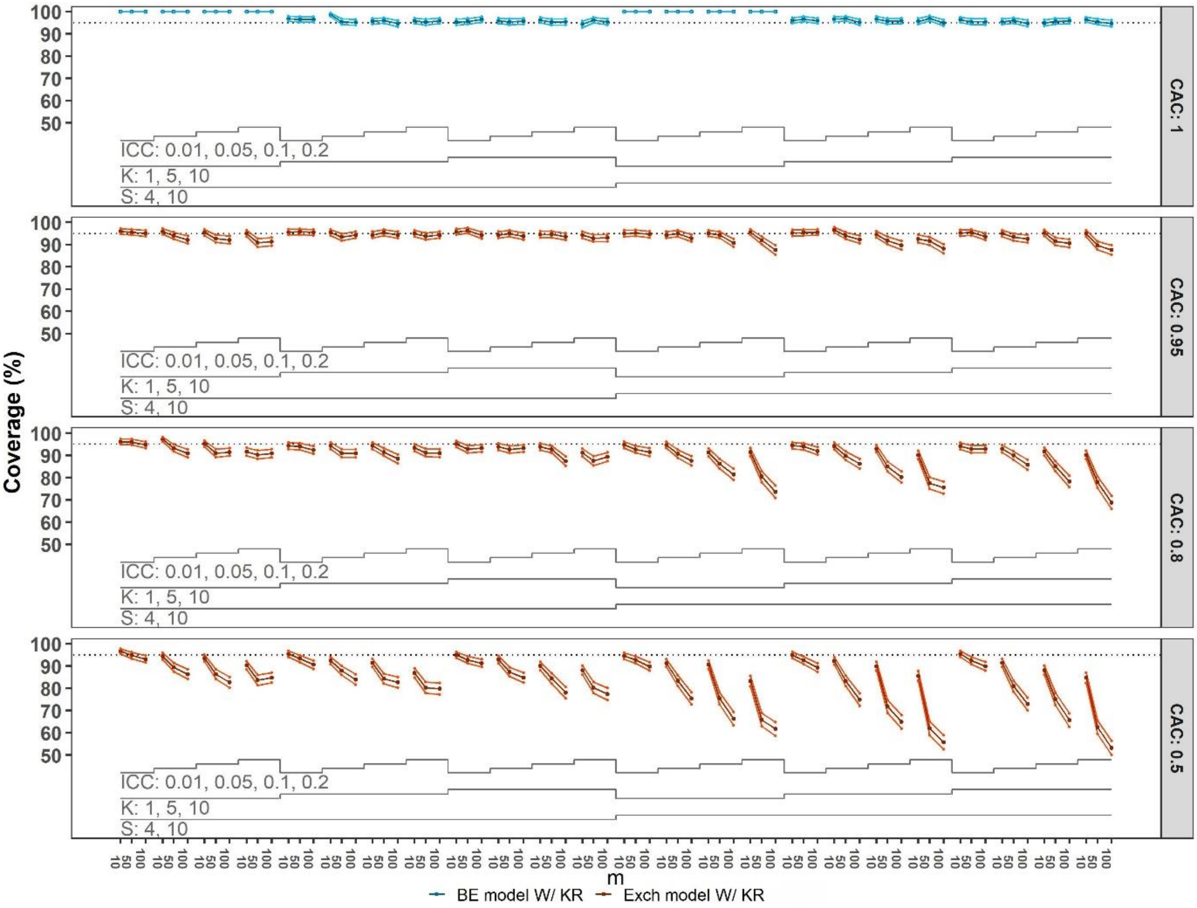

Figure $S7$. 95% Confidence interval coverage of overparameterised (top) and incorrectly specified models (all lower panels) with categorical time effects compared to the target coverage of 0.95 (dashed line). Monte Carlo standard error (MCSE) bounds of $\pm 2\times$ MCSE are included. For configurations with 50 or fewer clusters the Kenward-Roger (KR) small-sample correction method was used. Each panel corresponds to a CAC value: 1 (top), 0.95, 0.8, 0.5 (bottom). The panel includes the number of sequences $(S = 4, 10)$, the number of clusters per sequence $(K = 1, 5, 10)$, ICCs $(0.01, 0.05, 0.1, 0.2)$, and numbers of observations per cluster-period $(m = 10, 50, 100)$, yielding a total of 288 scenarios.

D Coverage with and without small-sample corrections

Satterthwaite small-sample correction

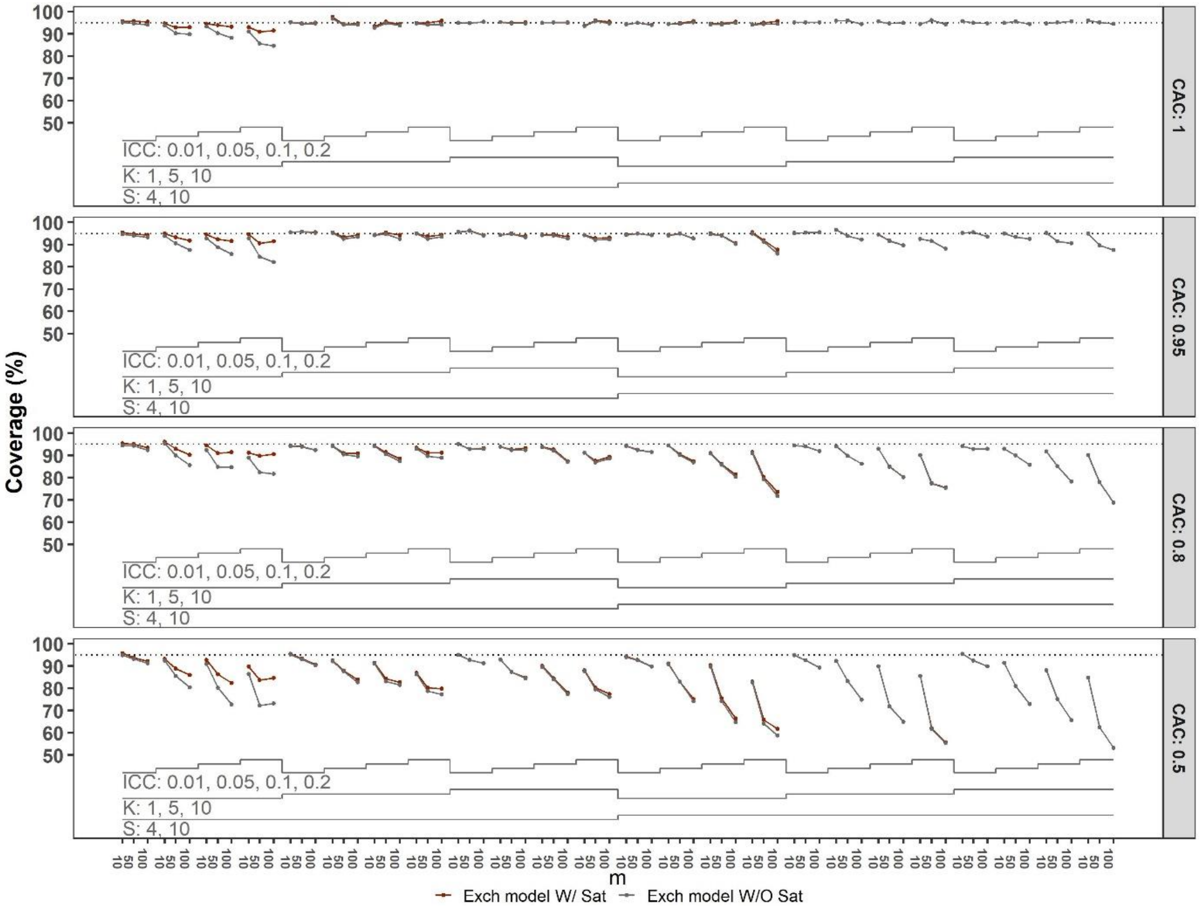

Figure $S8$. The coverage was calculated for the exchangeable (Exch) model with categorical time effects, with and without Satterthwaite (Sat) small-sample correction method. Each panel corresponds to a CAC value: 1 (top), 0.95, 0.8, 0.5 (bottom). The panel includes the number of sequences $(S = 4, 10)$, the number of clusters per sequence $(K = 1, 5, 10)$, ICCs $(0.01, 0.05, 0.1, 0.2)$, and numbers of observations per cluster-period $(m = 10, 50, 100)$, yielding a total of 288 scenarios for the exchangeable model without Satterthwaite small-sample correction and 240 scenarios for the exchangeable model with Satterthwaite small-sample correction.

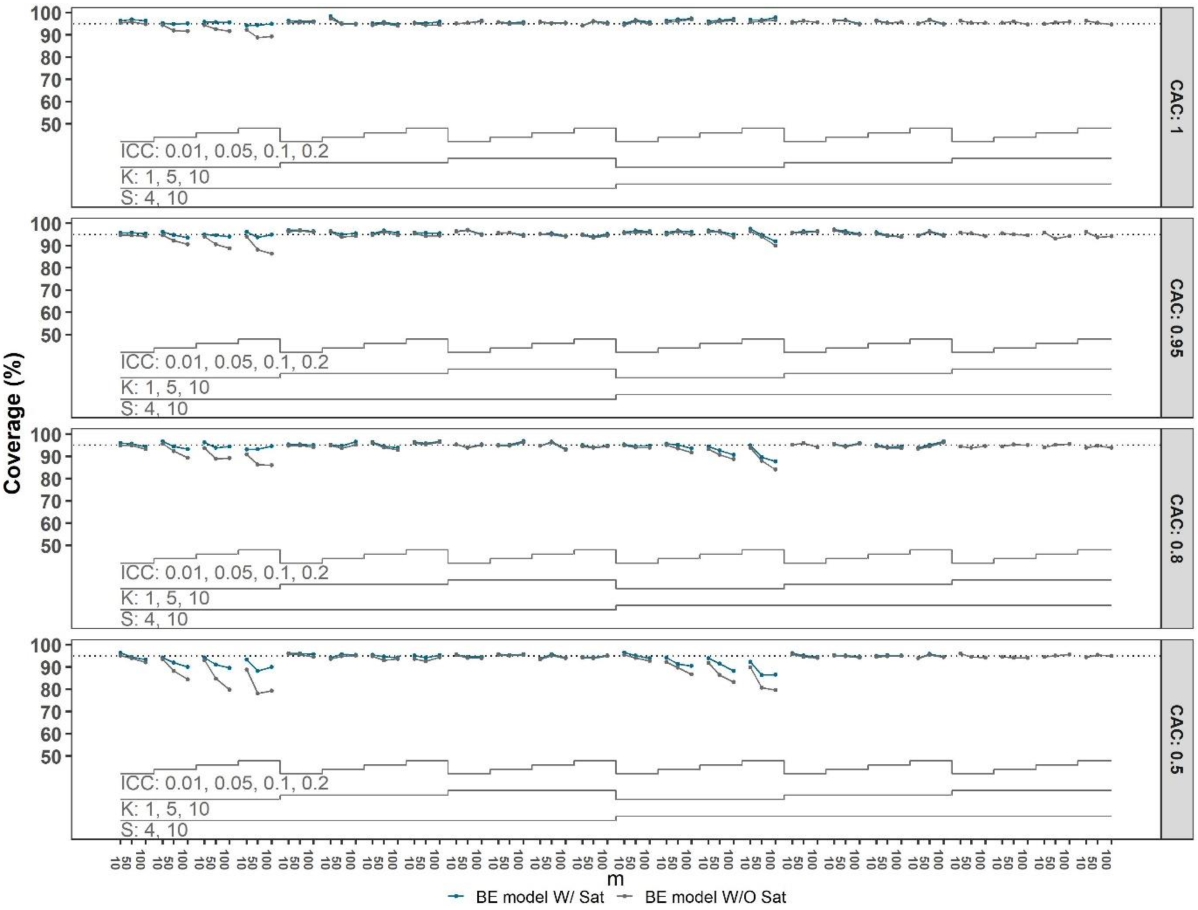

Figure $S9$. The coverage was calculated for the block-exchangeable (BE) model with categorical time effects, with and without Satterthwaite (Sat) small-sample correction method. Each panel corresponds to a CAC value: 1 (top), 0.95, 0.8, 0.5 (bottom). The panel includes the number of sequences $(S = 4, 10)$, the number of clusters per sequence $(K = 1, 5, 10)$, ICCs $(0.01, 0.05, 0.1, 0.2)$, and numbers of observations per cluster-period $(m = 10, 50, 100)$, yielding a total of 288 scenarios for the block-exchangeable model without Satterthwaite small-sample correction and 240 scenarios for the block-exchangeable model with Satterthwaite small-sample correction.

Kenward-Roger small-sample correction

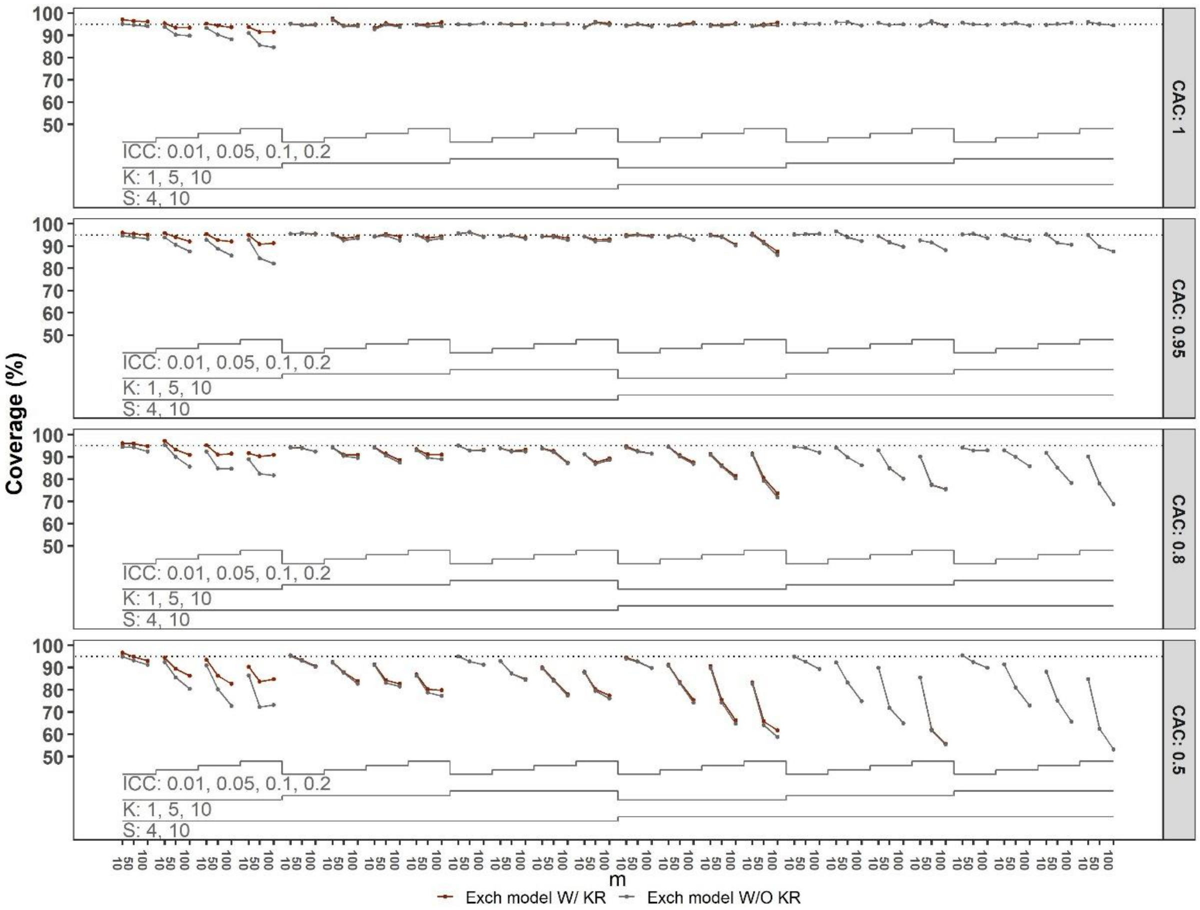

Figure $S10$. The coverage was calculated for the exchangeable (Exch) model with categorical time effects, with and without Kenward-Roger (KR) small-sample correction method. Each panel corresponds to a CAC value: 1 (top), 0.95, 0.8, 0.5 (bottom). The panel includes the number of sequences $(S = 4, 10)$, the number of clusters per sequence $(K = 1, 5, 10)$, ICCs $(0.01, 0.05, 0.1, 0.2)$, and numbers of observations per cluster-period $(m = 10, 50, 100)$, yielding a total of 288 scenarios for the exchangeable model without Kenward-Roger small-sample correction and 240 scenarios for the exchangeable model with Kenward-Roger small-sample correction.

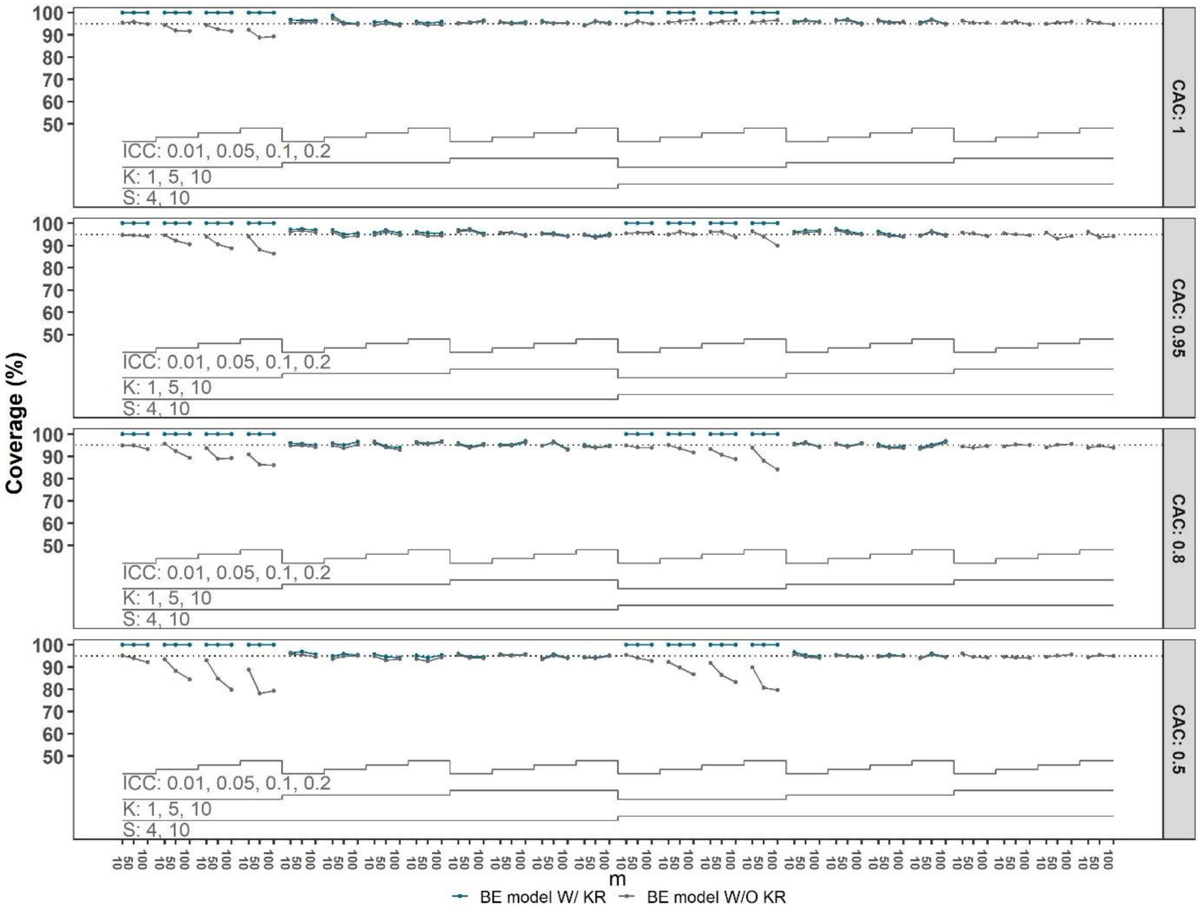

Figure $S11$. The coverage was calculated for the block-exchangeable (BE) model with categorical time effects, with and without Kenward-Roger (KR) small-sample correction method. Each panel corresponds to a CAC value: 1 (top), 0.95, 0.8, 0.5 (bottom). The panel includes the number of sequences $(S = 4, 10)$, the number of clusters per sequence $(K = 1, 5, 10)$, ICCs $(0.01, 0.05, 0.1, 0.2)$, and numbers of observations per cluster-period $(m = 10, 50, 100)$, yielding a total of 288 scenarios for the block-exchangeable model without Kenward-Roger small-sample correction and 240 scenarios for the block-exchangeable model with Kenward-Roger small-sample correction.

E Linear time effect

Type I error and power (Satterthwaite approximation)

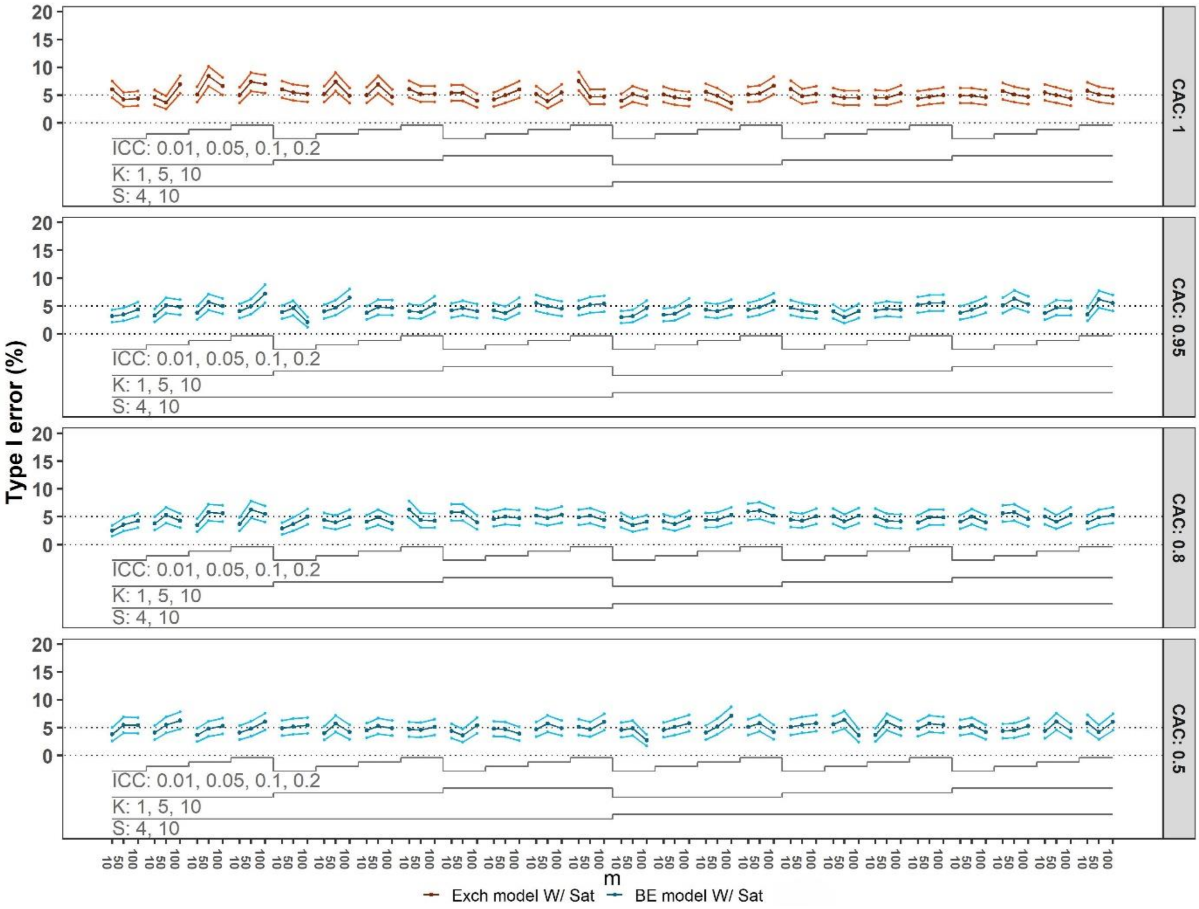

Figure $S12$. Type I error of correctly specified models with linear time effect compared to the nominal 5% level. Monte Carlo standard error (MCSE) bounds of $\pm 2 \times$ MCSE are included. For configurations with 50 or fewer clusters the Satterthwaite (Sat) small-sample correction method was used. Each panel corresponds to a CAC value: 1 (top), 0.95, 0.8, 0.5 (bottom). The panel includes the number of sequences $(S = 4, 10)$, the number of clusters per sequence $(K = 1, 5, 10)$, ICCs $(0.01, 0.05, 0.1, 0.2)$, and numbers of observations per cluster-period $(m = 10, 50, 100)$, yielding a total of 288 scenarios. BE=block-exchangeable; Exch=Exchangeable.

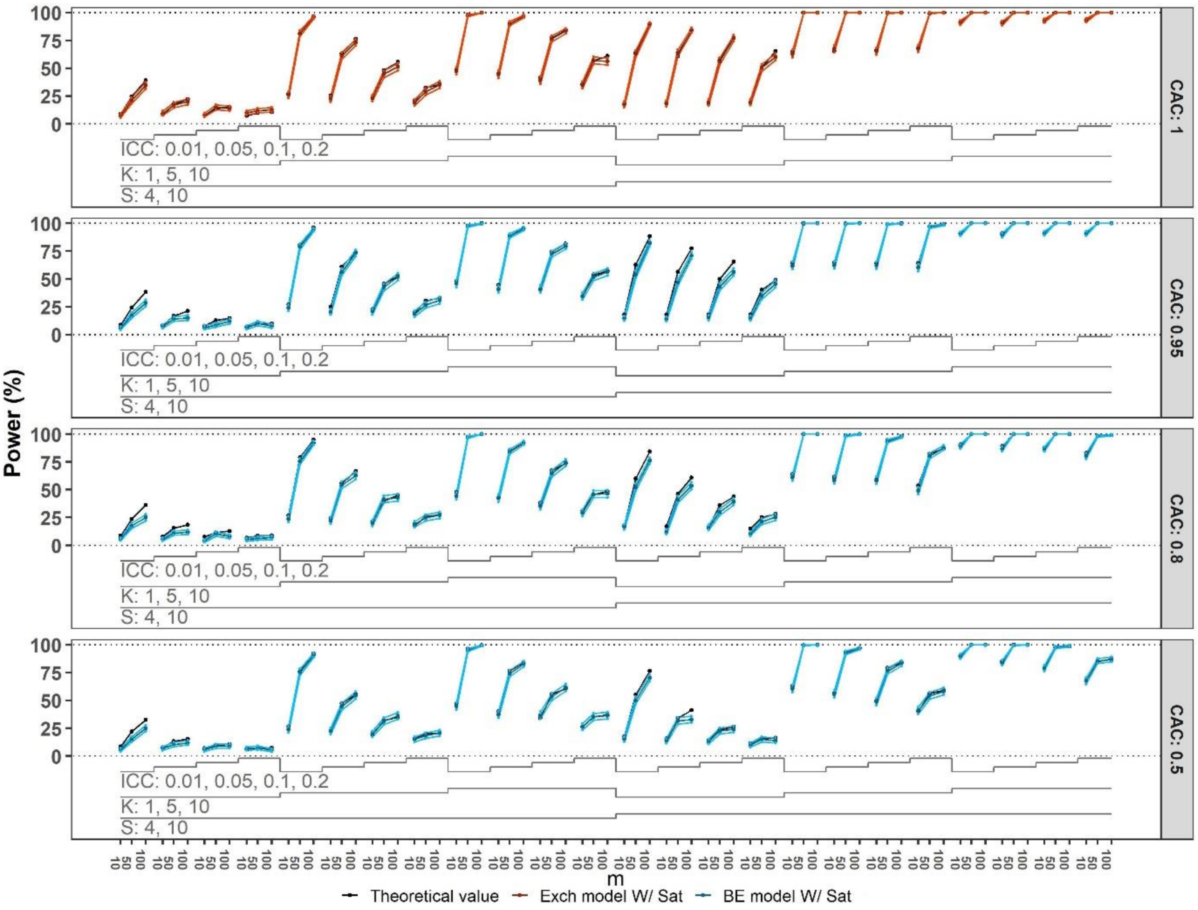

Figure $S13$. Power of correctly specified models with linear time effect compared to the theoretical value. Monte Carlo standard error (MCSE) bounds of $\pm 2 \times$ MCSE are included. For configurations with 50 or fewer clusters the Satterthwaite (Sat) small-sample correction method was applied. Each panel corresponds to a CAC value: 1 (top), 0.95, 0.8, 0.5 (bottom). The panel includes the number of sequences $(S = 4, 10)$, the number of clusters per sequence $(K = 1, 5, 10)$, ICCs $(0.01, 0.05, 0.1, 0.2)$, and numbers of observations per cluster-period $(m = 10, 50, 100)$, leading to a total of 288 scenarios. BE=block-exchangeable; Exch=Exchangeable.

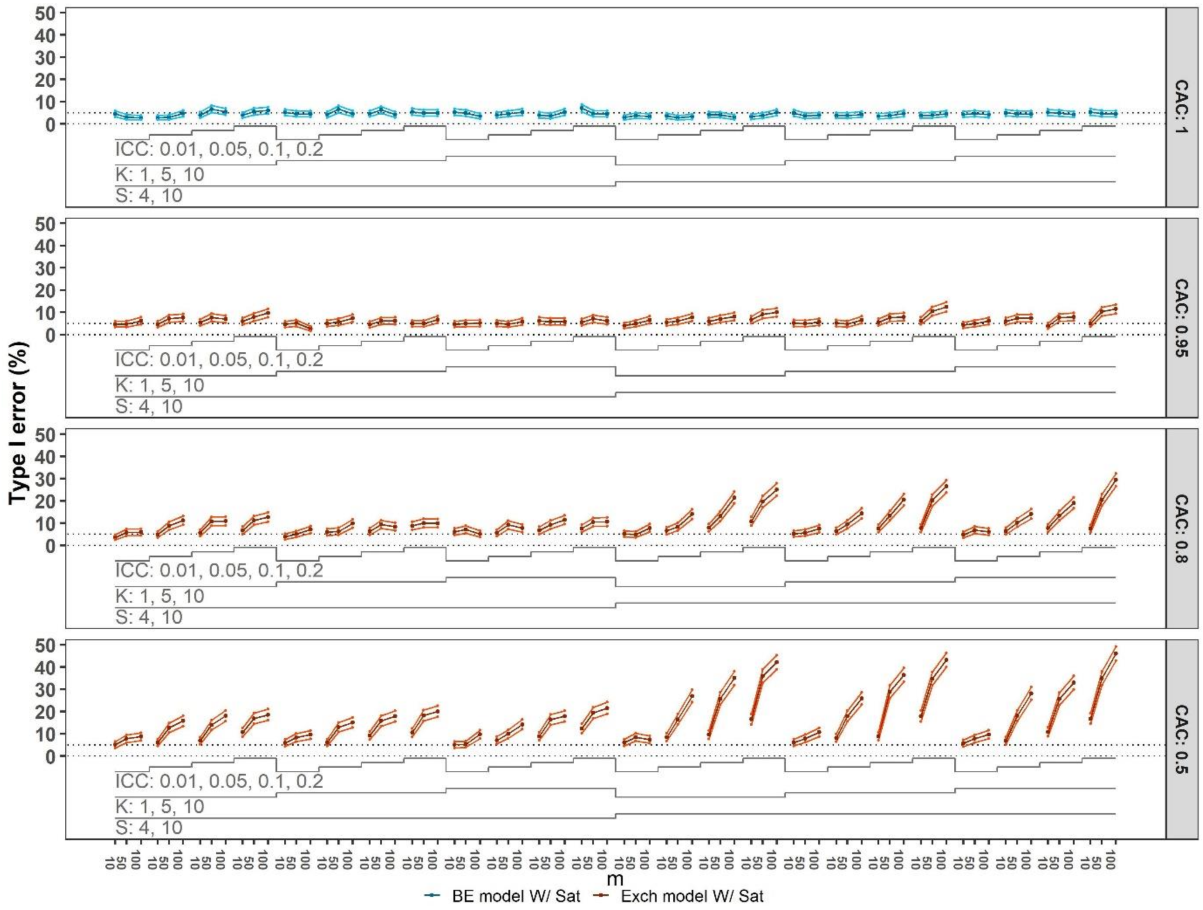

Figure $S14$. Type I error of overparameterised (top panel) and incorrectly specified models (all lower panels) with linear time effect compared to the nominal 5% value. Monte Carlo standard error (MCSE) bounds of $\pm 2 \times$ MCSE are included. For configurations with 50 or fewer clusters the Satterthwaite (Sat) small-sample correction method was used. Each panel corresponds to a CAC value: 1 (top), 0.95, 0.8, 0.5 (bottom). The panel includes the number of sequences ($S = 4, 10$), the number of clusters per sequence ($K = 1, 5, 10$), ICCs ($0.01, 0.05, 0.1, 0.2$), and numbers of observations per cluster-period ($m = 10, 50, 100$), yielding a total of 288 scenarios. BE=block-exchangeable; Exch=Exchangeable.

Coverage (Satterthwaite approximation)

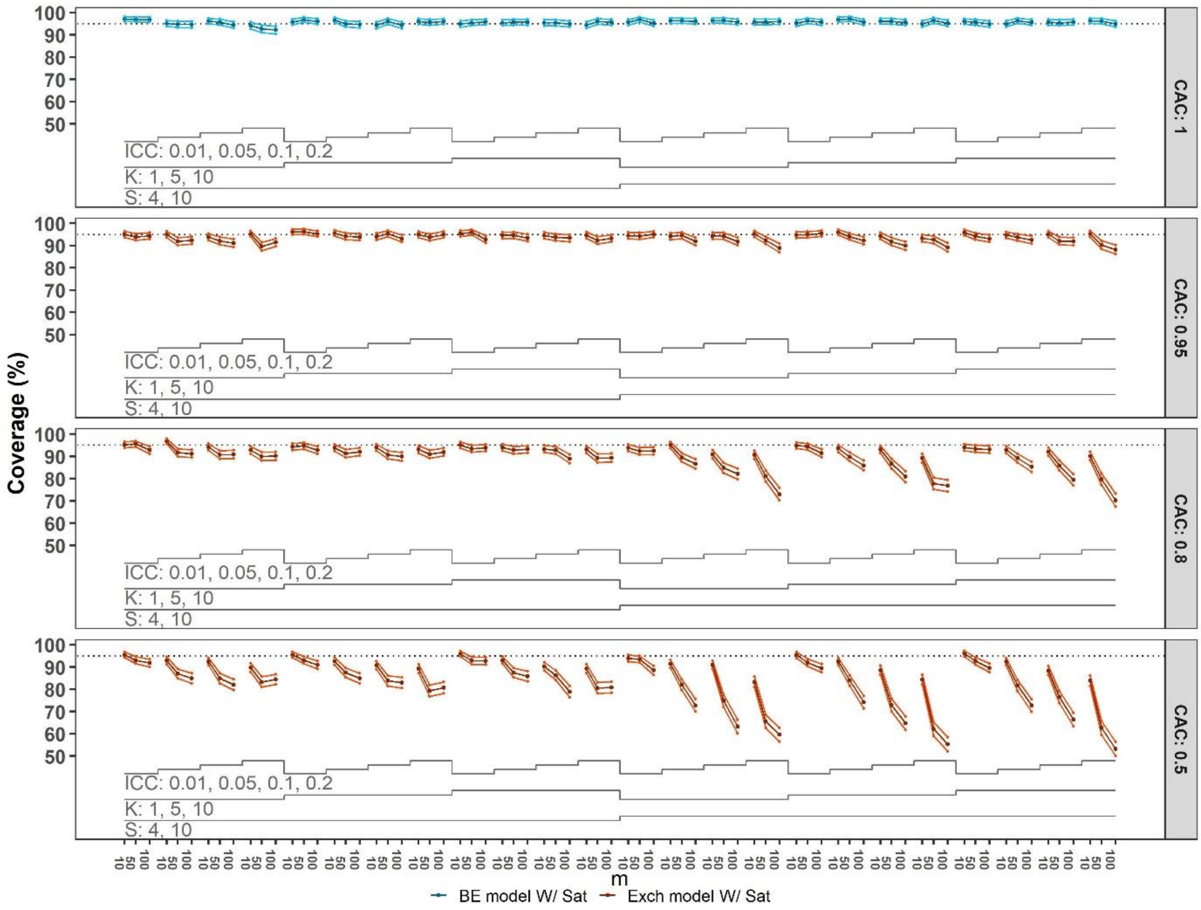

Figure $S15$. 95% Confidence interval coverage of overparameterised (top) and incorrectly specified models (all lower panels) with linear time effect compared to the target coverage of 0.95 (dashed line). Monte Carlo standard error (MCSE) bounds of $\pm 2 \times$ MCSE are included. For configurations with 50 or fewer clusters the Satterthwaite (Sat) small-sample correction method was used. Each panel corresponds to a CAC value: 1 (top), 0.95, 0.8, 0.5 (bottom). The panel includes the number of sequences ($S = 4, 10$), the number of clusters per sequence ($K = 1, 5, 10$), ICCs ($0.01, 0.05, 0.1, 0.2$), and numbers of observations per cluster-period ($m = 10, 50, 100$), yielding a total of 288 scenarios. BE=block-exchangeable; Exch=Exchangeable.

Type I error and power (Kenward-Roger approximation)

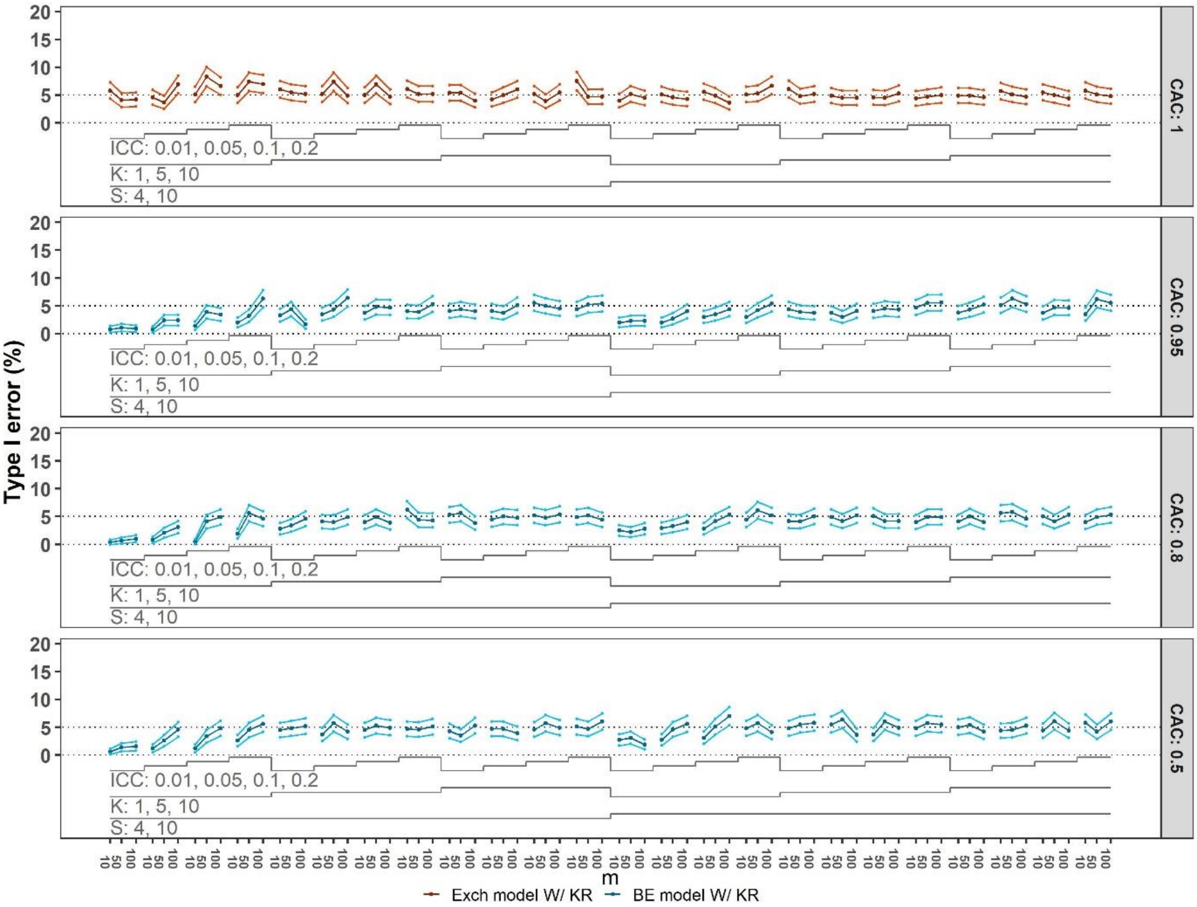

Figure $S16$. Type I error of correctly specified models with linear time effect compared to the nominal 5% level. Monte Carlo standard error (MCSE) bounds of $\pm\, 2 \times$ MCSE are included. For configurations with 50 or fewer clusters the Kenward-Roger (KR) small-sample correction method was used. Each panel corresponds to a CAC value: 1 (top), 0.95, 0.8, 0.5 (bottom). The panel includes the number of sequences $(S = 4, 10)$, the number of clusters per sequence $(K = 1, 5, 10)$, ICCs $(0.01, 0.05, 0.1, 0.2)$, and numbers of observations per cluster-period $(m = 10, 50, 100)$, yielding a total of 288 scenarios. BE=block-exchangeable; Exch=Exchangeable.

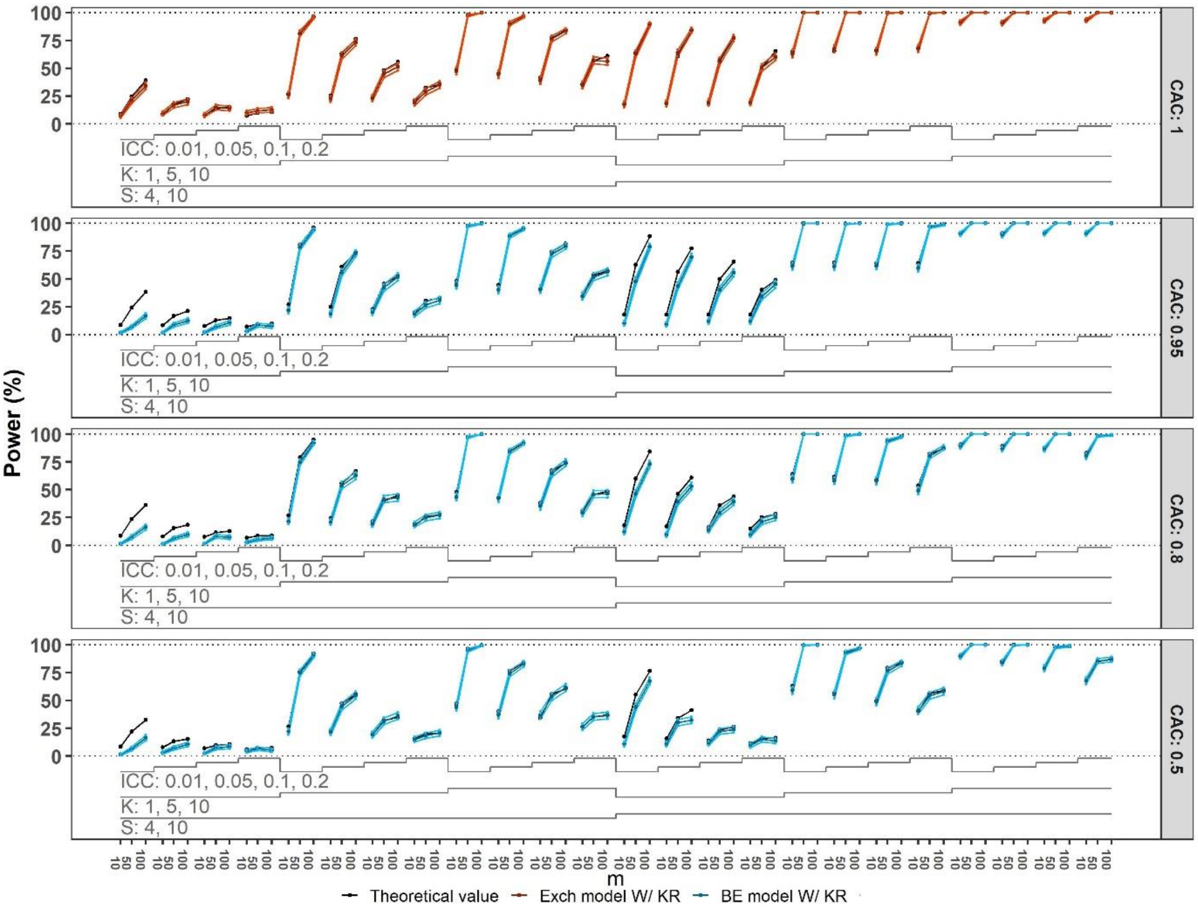

Figure $S17$. Power of correctly specified models with linear time effect compared to the theoretical value. Monte Carlo standard error (MCSE) bounds of $\pm 2 \times$ MCSE are included. For configurations with 50 or fewer clusters the Kenward-Roger (KR) small-sample correction method was applied. Each panel corresponds to a CAC value: 1 (top), 0.95, 0.8, 0.5 (bottom). The panel includes the number of sequences $(S = 4, 10)$, the number of clusters per sequence $(K = 1, 5, 10)$, ICCs $(0.01, 0.05, 0.1, 0.2)$, and numbers of observations per cluster-period ($m = 10, 50, 100$), leading to a total of 288 scenarios. BE=block-exchangeable; Exch=Exchangeable.

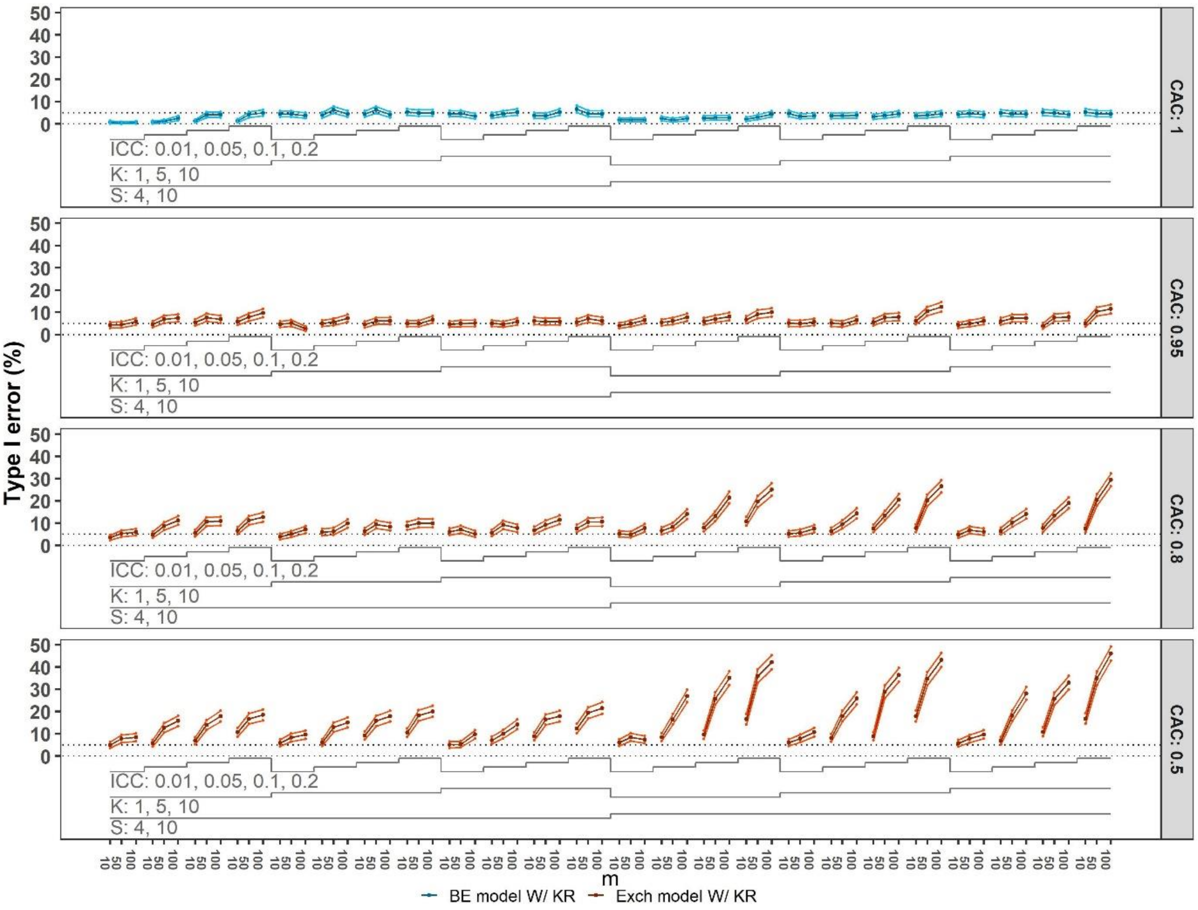

Figure $S18$: Type I error of overparameterised (top panel) and incorrectly specified models (all lower panels) with Linear time effect compared to the nominal 5% value. Monte Carlo standard error (MCSE) bounds of $\pm 2 \times$ MCSE are included. For configurations with 50 or fewer clusters the Kenward-Roger (KR) small-sample correction method was used. Each panel corresponds to a CAC value: 1 (top), 0.95, 0.8, 0.5 (bottom). The panel includes the number of sequences ($S = 4, 10$), the number of clusters per sequence ($K = 1, 5, 10$), ICCs ($0.01, 0.05, 0.1, 0.2$), and numbers of observations per cluster-period ($m = 10, 50, 100$), yielding a total of 288 scenarios. BE=block-exchangeable; Exch=Exchangeable.

Coverage (Kenward-Roger approximation)

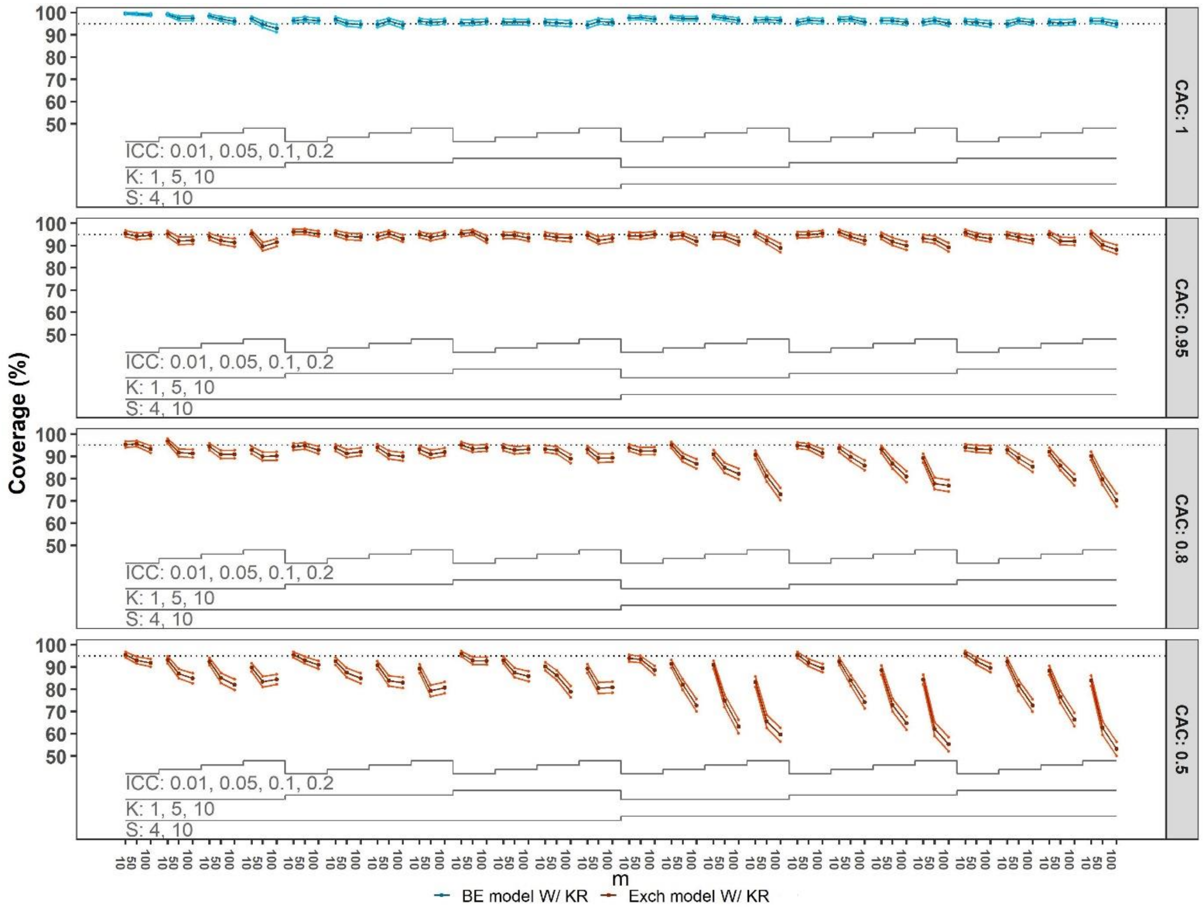

Figure $S19$. 95% Confidence interval coverage of overparameterised (top) and incorrectly specified models (all lower panels) with linear time effect compared to the target coverage of 0.95 (dashed line). Monte Carlo standard error (MCSE) bounds of $\pm 2 \times$ MCSE are included. For configurations with 50 or fewer clusters the Kenward-Roger (KR) small-sample correction method was used. Each panel corresponds to a CAC value: 1 (top), 0.95, 0.8, 0.5 (bottom). The panel includes the number of sequences $(S = 4, 10)$, the number of clusters per sequence $(K = 1, 5, 10)$, ICCs $(0.01, 0.05, 0.1, 0.2)$, and numbers of observations per cluster-period $(m = 10, 50, 100)$, yielding a total of 288 scenarios. BE=block-exchangeable; Exch=Exchangeable.

Bias for treatment effect

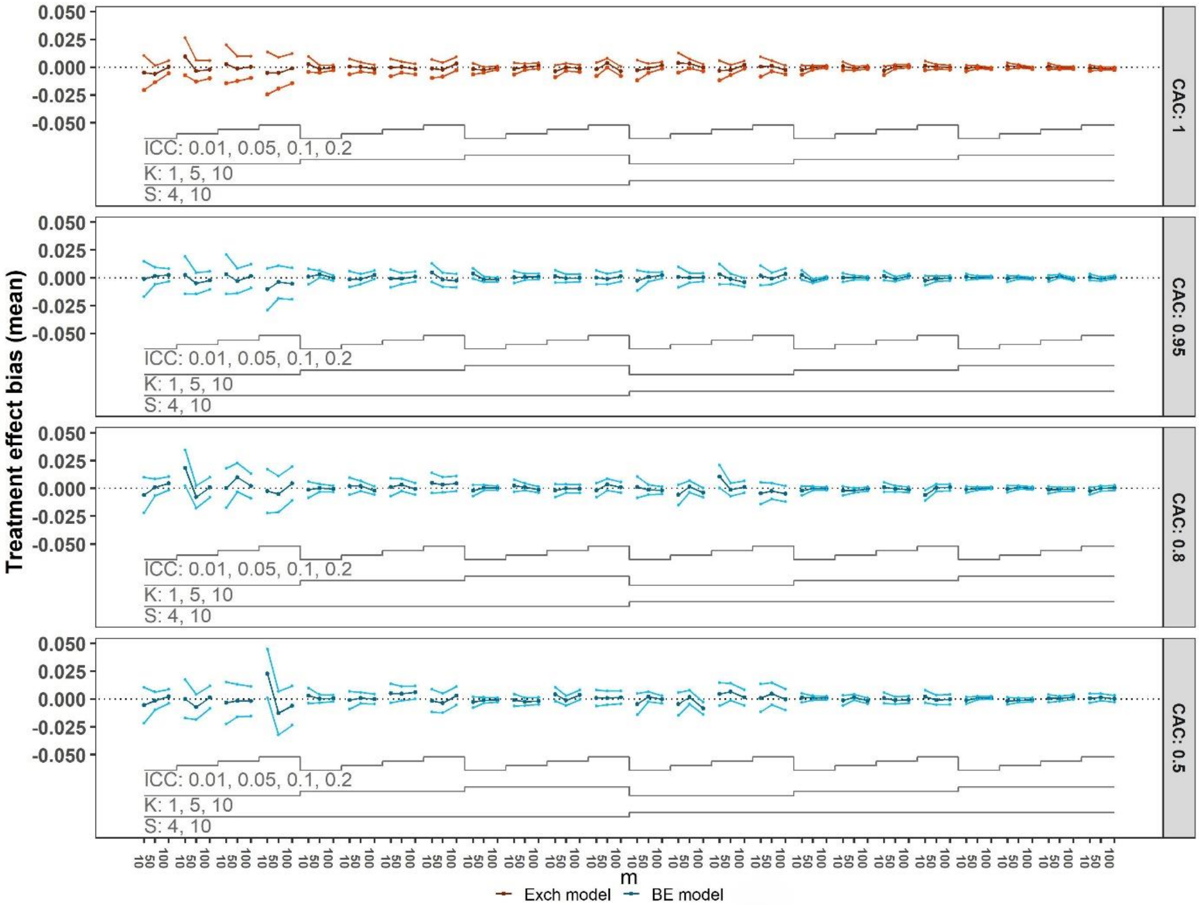

Figure $S20$. Bias for treatment effect of correctly specified models with linear time effect. Monte Carlo standard error (MCSE) bounds of $\pm 2 \times$ MCSE are included. Each panel corresponds to a CAC value: 1 (top), 0.95, 0.8, 0.5 (bottom). The panel includes the number of sequences ($S = 4, 10$), the number of clusters per sequence ($K = 1, 5, 10$), ICCs ($0.01, 0.05, 0.1, 0.2$), and numbers of observations per cluster-period ($m = 10, 50, 100$), yielding a total of 288 scenarios. BE=block-exchangeable; Exch=Exchangeable.

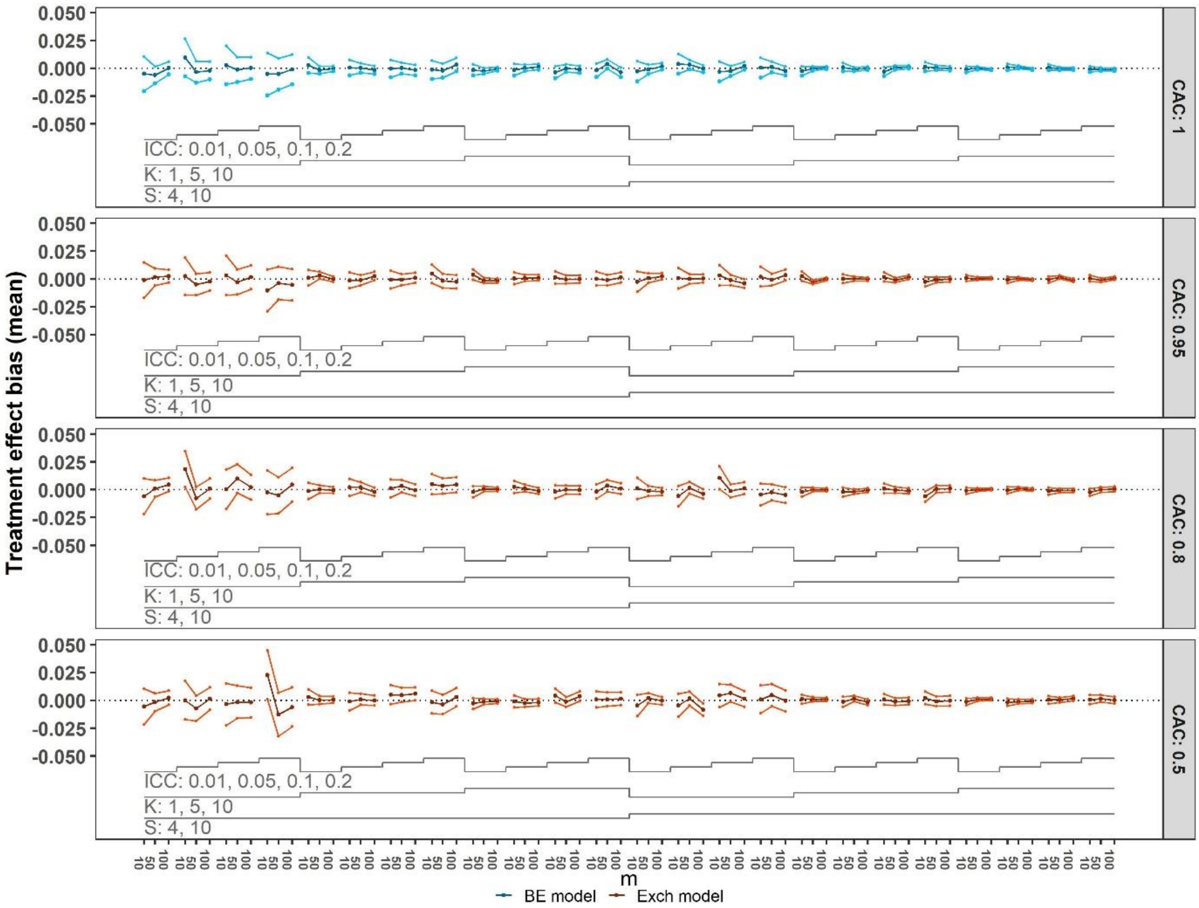

Figure $S21$. Bias for treatment effect of overparameterised (top panel) and incorrectly specified models (all lower panels) with linear time effect. Monte Carlo standard error (MCSE) bounds of $\pm 2 \times$ MCSE are included. Each panel corresponds to a CAC value: 1 (top), 0.95, 0.8, 0.5 (bottom). The panel includes the number of sequences ($S = 4, 10$), the number of clusters per sequence ($K = 1, 5, 10$), ICCs $(0.01, 0.05, 0.1, 0.2)$, and numbers of observations per cluster-period ($m = 10, 50, 100$), yielding a total of 288 scenarios. BE=block-exchangeable; Exch=Exchangeable.
